# Aerobic alcohol oxidation and oxygen atom transfer reactions catalyzed by a nonheme iron(ii)–α-keto acid complex†
†Electronic supplementary information (ESI) available: Crystallographic data in CIF format and spectroscopic data. CCDC 1061509 and 1061973. For ESI and crystallographic data in CIF or other electronic format see DOI: 10.1039/c6sc01476c


**DOI:** 10.1039/c6sc01476c

**Published:** 2016-04-25

**Authors:** Debobrata Sheet, Tapan Kanti Paine

**Affiliations:** a Department of Inorganic Chemistry , Indian Association for the Cultivation of Science , 2A & 2B Raja S. C. Mullick Road, Jadavpur , Kolkata 700032 , India . Email: ictkp@iacs.res.in ; Fax: +91-33-2473-2805 ; Tel: +91-33-2473-4971

## Abstract

α-Ketoglutarate-dependent enzymes catalyze many important biological oxidation/oxygenation reactions. Iron(iv)–oxo intermediates have been established as key oxidants in these oxidation reactions. While most reported model iron(ii)–α-keto acid complexes exhibit stoichiometric reactivity, selective oxidation of substrates with dioxygen catalyzed by biomimetic iron(ii)–α-keto acid complexes remains unexplored. In this direction, we have investigated the ability of an iron(ii) complex [(Tp^Ph,Me^)Fe^II^(BF)] (**1**) (Tp^Ph,Me^ = hydrotris(3-phenyl-5-methylpyrazolyl)borate and BF = monoanionic benzoylformate) to catalyze the aerobic oxidation of organic substrates. An iron–oxo oxidant, intercepted in the reaction of **1** with O_2_, selectively oxidizes sulfides to sulfoxides, alkenes to epoxides, and alcohols to the corresponding carbonyl compounds. The oxidant from **1** is able to hydroxylate the benzylic carbon of phenylacetic acid to afford mandelic acid with the incorporation of one oxygen atom from O_2_ into the product. The iron(ii)–benzoylformate complex oxidatively converts phenoxyacetic acids to the corresponding phenols, thereby mimicking the function of iron(ii)–α-ketoglutarate-dependent 2,4-dichlorophenoxyacetate dioxygenase (TfdA). Furthermore, complex **1** exhibits catalytic aerobic oxidation of alcohols and oxygen atom transfer reactions with multiple turnovers.

## Abstract 2

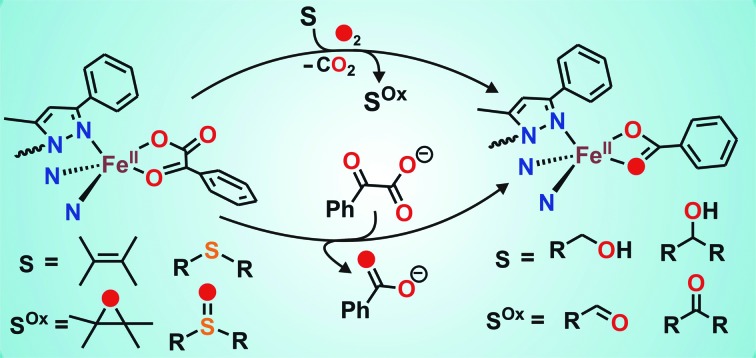
An iron(ii)-benzoylformate complex of a monoanionic facial tridentate ligand catalyzes the aerobic oxidation of sulfides to sulfoxides, alkenes to epoxides, and alcohols to the corresponding carbonyl compounds.

## Introduction

α-Ketoglutarate-dependent enzymes represent the largest subfamily of nonheme iron enzymes and catalyze a myriad of biological oxidation reactions.^1^ These enzymes are involved in important biological processes such as the hydroxylation of protein side chains, DNA/RNA repair, biosynthesis of antibiotics, lipid metabolism, biodegradation of toxic compounds, transcription regulation, and oxygen sensing.^1^–^3^ Despite the diversity of reactions, all the enzymes in this subfamily have a common ‘2-His-1-carboxylate facial triad’ motif.^3^,^4^ These enzymes require an α-ketoglutarate cofactor for the reductive activation of dioxygen and subsequent generation of iron–oxygen oxidant.^3^,^5^ In the reactions, the metal-bound α-ketoglutarate cofactor undergoes oxidative decarboxylation to form carbon dioxide and succinate. On the basis of a large number of crystallographic,^6^,^7^ spectroscopic,^8^–^10^ and DFT studies,^10^ a common mechanism of α-ketoglutarate-dependent enzymes has been put forward.^1^,^2^,^7^,^11^ Initial binding of dioxygen to the iron(ii) center of the enzyme leads to the formation of an iron(iii)–superoxo species. The nucleophilic superoxide then attacks the electrophilic keto carbon of the coordinated α-KG to form an iron(iv)–alkylperoxo species. The peroxo species undergoes O–O and C–C bond cleavage to generate CO_2_, succinate, and iron(iv)–oxo intermediate. Nonheme iron(iv)–oxo intermediates have been identified as active oxidants in the catalytic cycles of a number of α-keto acid-dependent enzymes.^5^,^12^–^18^ Biomimetic iron(ii)–α-keto acid complexes have also provided useful information about the mechanism of α-ketoglutarate-dependent enzymes.^19^–^23^ *In situ* generated iron(iv)–oxo species, intercepted in the reactions between iron(ii)–α-keto acid complexes of polydentate supporting ligands and dioxygen, have been reported to exhibit stoichiometric reactivity toward alkenes, alkanes, sulfides and alcohols.^24^ Among the reported models, the iron(ii)–α-keto acid complexes of tris(pyrazolyl)borate ligands represent the most efficient functional models of the α-ketoglutarate dependent enzymes. Monoanionic tris(pyrazolyl)borates provide a facial N_3_ ligand environment at the metal center and mimic the ‘2-His-1-carboxylate facial triad’ motif observed in α-ketoglutarate dependent enzymes.^25^ Moreover, the steric and electronic properties of these ligands can be easily tuned by placing appropriate substituents on the pyrazole rings.^26^ The iron(ii)–benzoylformate complex [(Tp^Me2^)Fe^II^(BF)] (BF = monoanionic benzoylformate) represents the first biomimetic complex of a facial N_3_ ligand, which reacts with oxygen within 2 minutes to generate an oxidant capable of epoxidizing cyclohexene and *cis*-stilbene.^27^ For *cis*-stilbene, the retention of configuration in the epoxide product indicates the involvement of a metal-based oxidant. The complex, however, cannot oxidize *trans*-stilbene, suggesting that the oxidant generated is capable of discriminating between the *cis* and *trans* isomers of an olefin.^27^ While the iron(ii)–α-keto acid complex of Tp^3tBu,5iPr^ is unreactive toward dioxygen,^28^ those of Tp^iPr2^ and Tp^Ph2^ ligands^21^,^23^,^29^ react with O_2_ to exhibit oxidative decarboxylation. Compared to the Tp^Me2^ system, iron(ii)–α-keto acid complexes of Tp^iPr2^ or Tp^Ph2^ display slower reactivity toward dioxygen. The accessibility of dioxygen to the iron center depends on the steric bulk engendered by the substituent on pyrazole which in turn decides the reactivity with dioxygen.

**Scheme 1:**
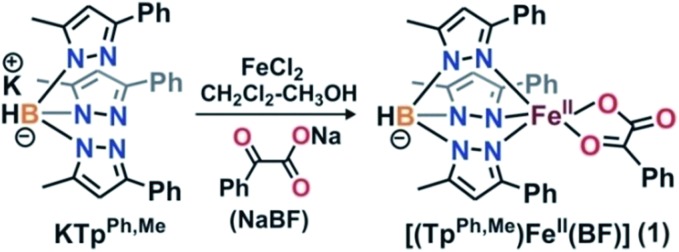
Synthesis of iron(ii)–benzoylformate complex.

With an objective to develop iron-based catalysts for the selective oxidation of organic substrates with O_2_, we have investigated the reactivity of iron(ii) complexes supported by a hydrotris(3-phenyl-5-methylpyrazolyl)borate (Tp^Ph,Me^) ligand in the presence of different co-substrates as ligands.^30^ The co-ligands provide the necessary electrons for dioxygen reduction to generate a metal–oxygen oxidant which can oxidize organic substrates. As a part of our investigation, we report herein the synthesis, characterization and dioxygen reactivity of a biomimetic iron(ii)–α-keto acid complex, [(Tp^Ph,Me^)Fe^II^(BF)] (**1**) (Scheme 1). In a reaction with dioxygen, the iron(ii) complex selectively oxidizes alcohols to ketones, alkenes to epoxides, and sulfides to sulfoxides using dioxygen as the oxidant. The interception of a high-valent iron–oxo oxidant from O_2_, and catalytic and mechanistic studies of alcohol oxidation and oxo-atom transfer reactions by complex **1**, are presented in this article.

## Results and discussion

Complex [(Tp^Ph,Me^)Fe^II^(BF)] (**1**) was isolated from the reaction of equimolar amounts of KTp^Ph,Me^, iron(ii) chloride, and sodium benzoylformate (NaBF) in a CH_2_Cl_2_–CH_3_OH solvent mixture (see Experimental section and Scheme 1). The ESI mass spectrum of complex **1** displays a molecular ion peak at *m*/*z* = 689.1 with an isotope distribution pattern attributable to {[(Tp^Ph,Me^)Fe(BF)] + H}^+^ (Fig. S1, ESI†). In the ^1^H NMR spectrum, the complex displays paramagnetically shifted resonances of protons as observed with high-spin iron(ii) complexes of the ligand (Fig. S2, ESI†).^30^ The optical spectrum of the complex in acetonitrile exhibits broad absorption bands at 537 nm (315 M^–1^ cm^–1^) and 580 nm (300 M^–1^ cm^–1^) suggesting a chelating bidentate coordination of the α-keto acid (BF) anion.^19^ Although the intensities of absorption bands are low in **1**, similar absorption features are observed in iron(ii)–benzoylformate complexes of tris(pyrazolyl)borate ligands.^21^,^27^,^28^ The origin of the absorption bands may be attributed to the MLCT transitions from Fe(ii)-to-π* orbital of the keto group.^8^

**Fig. 1:**
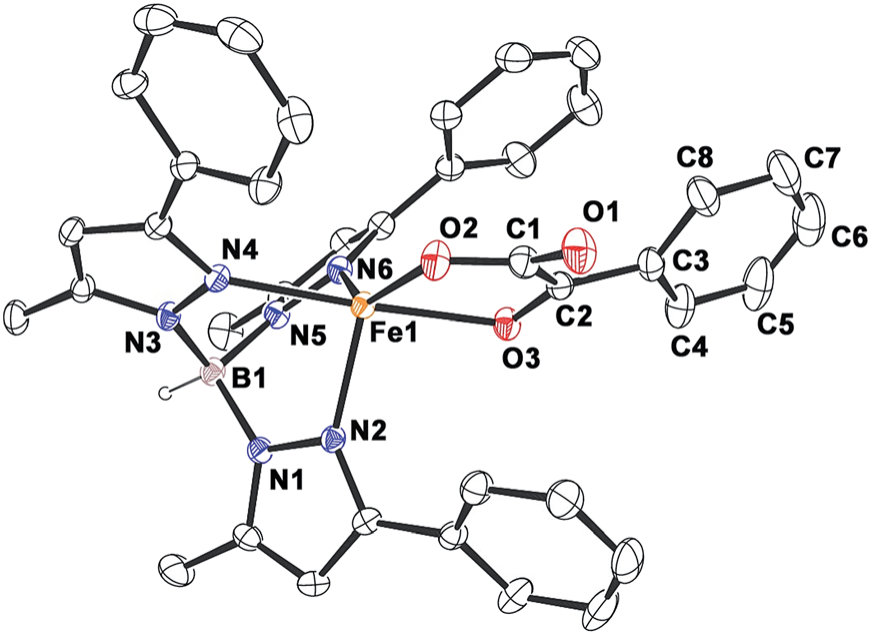
ORTEP plot of [(Tp^Ph,Me^)Fe^II^(BF)] (**1**). All the hydrogen atoms except for B1 have been omitted for clarity. Selected bond lengths [Å] and angles [°] for **1**: Fe1–N2 2.083(2), Fe1–N4 2.209(2), Fe1–N6 2.065(2), Fe1–O2 1.957(2), Fe1–O3 2.262(2), N4–Fe1–O3 172.98(7), N6–Fe1–O2 138.42(9), N6–Fe1–N2 92.41(8), N6–Fe1–O3 87.02(6), N4–Fe1–N6 86.13(7), N4–Fe1–N2 89.50(7), N2–Fe1–O3 92.32(7), N4–Fe1–O2 109.26(7), O2–Fe1–O3 75.13(6), and N2–Fe1–O2 124.95(8).

The complex was further characterized by single crystal X-ray diffraction studies. The X-ray structure of the neutral complex reveals that the iron center is coordinated by a tridentate monoanionic face-capping Tp^Ph,Me^ ligand and a bidentate monoanionic benzoylformate (Fig. 1). Benzoylformate is coordinated to the iron center *via* a carboxylate oxygen (O2) and the carbonyl oxygen (O3) with the Fe1–O2 and the Fe1–O3 distances of 1.957(2) Å and 2.262(2) Å, respectively. The M–O distances are comparable to those reported for five-coordinate iron(ii)–α-keto acid complexes of Tp^R1,R2^ type ligands.^21^,^28^,^30^ The average iron(ii)–N(pyrazole) bond length is typical of high-spin iron(ii) complexes of tris(pyrazolyl)borate ligands. The geometry of the five-coordinate iron complex can be best described as distorted trigonal bipyramidal (*τ* = 0.57)^31^ with the equatorial plane being formed by the carboxylate oxygen O2, and the pyrazole nitrogens N6 and N2. The pyrazole nitrogen N4 and the keto oxygen O3 occupy the axial positions with an N4–Fe1–O3 angle of 172.98(7)° (Table S1†). Interestingly, the Fe–O(keto) and the Fe–N4 bonds are significantly elongated compared to those in complex [(Tp^Ph2^)Fe^II^(BF)].^21^

**Fig. 2:**
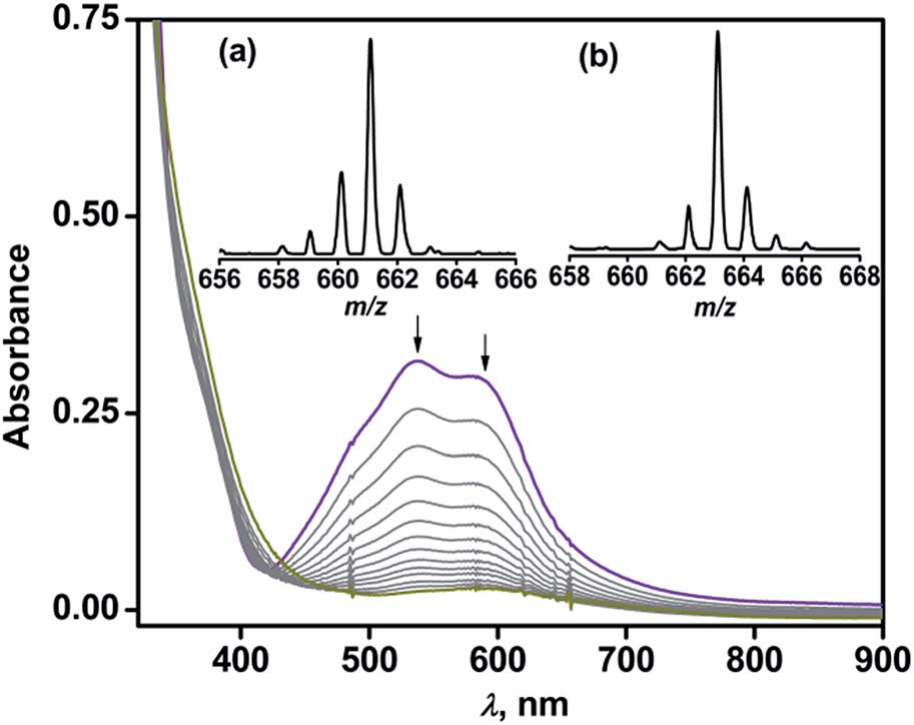
Optical spectral changes during the reaction of **1** (1 mM) with dioxygen in acetonitrile at 298 K. Inset: ESI mass spectra of the oxidized solution after the reaction of **1** with (a) ^16^O_2_ and (b) ^18^O_2_ in acetonitrile.

Complex **1** reacts with dioxygen in acetonitrile over a period of 1.5 h, during which the violet solution turns colorless. In the reaction, the MLCT bands at 537 nm and 580 nm decay following a pseudo-first order rate (*k*_obs_ = 5.5 × 10^–4^ s^–1^) to yield an almost featureless optical spectrum, indicating the oxidative decarboxylation of coordinated BF (Fig. 2). Contrary to the results obtained with [(Tp^Ph2^)Fe^II^(BF)],^21^,^29^ intra-ligand hydroxylation is not observed in the reaction of complex **1** with O_2_. The ESI-MS of the oxidized solution of **1** shows an ion peak at *m*/*z* = 661.2 with the isotope distribution pattern calculated for {[(Tp^Ph,Me^)Fe(OBz)] + H}^+^ (OBz = benzoate). When the reaction is carried out with ^18^O_2_, the ion peak is shifted two mass units higher to *m*/*z* = 663.2 (Fig. 2 inset). The ^1^H NMR spectrum of the final reaction solution bears resemblance to that of an independently prepared iron(ii)–benzoate complex [(Tp^Ph,Me^)Fe^II^(OBz)] (**2**) (see Experimental section, and Scheme S1 and Fig. S2, ESI†). The oxidized solution of **1** is X-band EPR silent at 77 K, further indicating the formation of an iron(ii) complex. Time-dependent ^1^H NMR spectra after the removal of the metal ions establish the complete decarboxylation of BF to benzoic acid in about 1.5 h (Fig. S3, ESI†). The conversion of BF to benzoic acid by the iron(ii) complex with the incorporation of one oxygen atom from O_2_ into benzoate functionally mimics the reaction catalyzed by α–keto acid-dependent oxygenases.

**Scheme 2:**
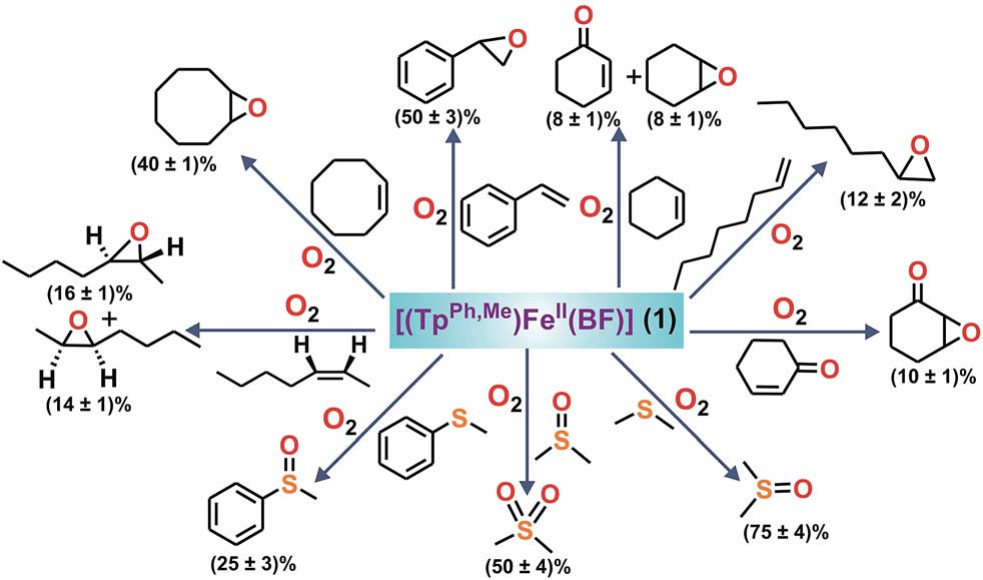
Oxidation of various substrates by complex **1**.

To intercept the iron–oxygen intermediate from **1**, external substrates were used as indirect probes (Scheme 2).^29^,^32^,^33^ Reaction of **1** with O_2_ in the presence of thioanisole (10 equiv.) affords 25% thioanisole oxide as the only product (Fig. S4, ESI†). A comparatively higher amount of interception is observed with smaller substrates such as dimethyl sulfide (DMS) and dimethyl sulfoxide (DMSO). While DMSO is formed from DMS in about 75% yield, around 50% dimethyl sulfone is formed from DMSO (Fig. S5 and S6, ESI†). On the other hand, relatively bulky dibenzothiophene could intercept the active oxidant to an extent of only 6%.

To explore the reactivity of the oxidant further, reactions were carried out between **1** and various alkenes (Scheme 2). Styrene (100 equiv.) is found to be the most effective alkene substrate affording styrene epoxide (50%). While *trans*-2-heptene exclusively forms *trans*-epoxide in 25% yield, the oxidation of *cis*-2-heptene affords a mixture of isomers, *cis*-2-heptene oxide (14%) and *trans*-2-heptene oxide (16%) (Fig. S7, ESI†). The electron deficient alkene 2-cyclohexenone (100 equiv.) forms about 10% 2-cyclohexenone oxide (Fig. S8, ESI†), but *tert*-butyl acrylate and dimethyl fumarate are not epoxidized under similar experimental conditions. A low yield is obtained with 1-octene, forming only 12% epoxide product (Fig. S9, ESI†). With cyclooctene as the substrate, cyclooctene oxide (40%) is obtained as the exclusive product without any diol (Fig. S10, ESI†). Cyclohexene (100 equiv.) gives cyclohexene oxide (8%) along with the allylic oxidation products 2-cyclohexen-1-ol (2%) and 2-cyclohexene-1-one (8%) (Fig. S11, ESI†).

The ability of complex **1** to cleave an aliphatic C–H bond was studied (Scheme S2, ESI†). Substrates with relatively weaker C–H bonds such as fluorene (10 equiv.) and 9,10-dihydranthracene (10 equiv.) intercept the active oxidant from **1** with the formation of 40% fluorenone, and 45% anthracene with a trace amount of anthraquinone, respectively (Fig. S12 and S13, ESI†). Lower yields are obtained using hydrocarbons with relatively stronger C–H bonds. While ethylbenzene (100 equiv.) affords a mixture of acetophenone (5%) and 1-phenylethanol (4%), adamantane (10 equiv.) gets oxidized to a mixture of 1-adamantanol (∼10%), 2-adamantanol (∼12%) and 2-adamantanone (∼2%) (Scheme S2, Fig. S14 and S15, ESI†). Toluene and cyclohexane are not oxidized, suggesting that the oxidant from **1** is not powerful enough to cleave the stronger C–H bonds.

**Fig. 3:**
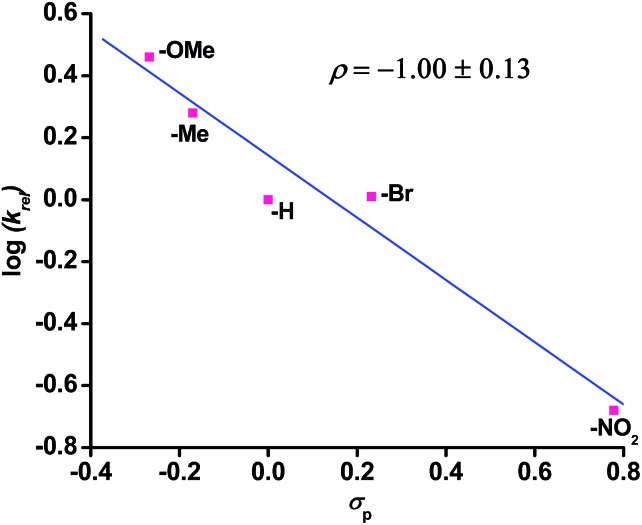
Hammett plot of log *k*_rel_*vs. σ*_p_ for *p*-C_6_H_4_–SMe. The *k*_rel_ values were calculated by dividing the concentration of product from *para*-substituted thioanisole by the concentration of product from thioanisole.

The reaction of complex **1** with dioxygen gives the activation parameters of Δ*H*^≠^ = 27 kJ mol^–1^ and Δ*S*^≠^ = –216 J mol^–1^ K^–1^ determined from Eyring analysis (Fig. S16, ESI†). The large negative entropy of activation indicates that the rate determining step is associative in nature. Hammett analyses from competitive reactions using equimolar amounts of a *para*-substituted thioanisoles and thioanisole with complex **1** and dioxygen reveal a negative *ρ* value of –1.00 (±0.13) supporting an electrophilic oxidant being responsible for sulfide oxidation (Fig. 3).

**Scheme 3:**
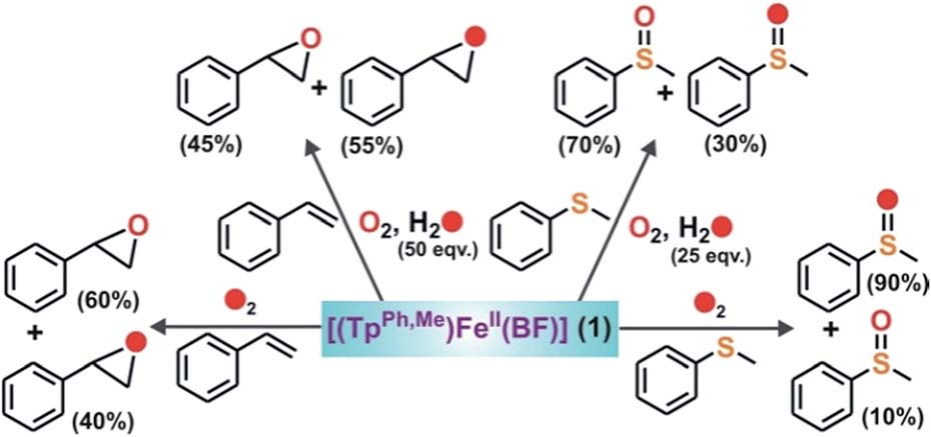
Incorporation of labelled oxygen in the oxidation of styrene and thioanisole by **1**.

**Fig. 4:**
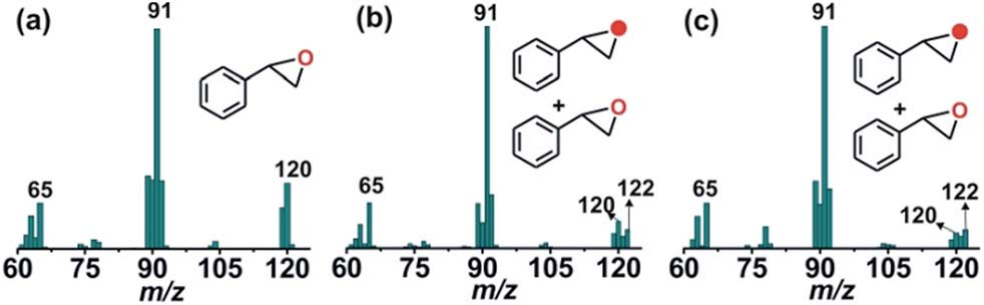
GC-mass spectra of styrene oxide formed from styrene in the reaction of **1** with (a) ^16^O_2_, (b) ^18^O_2_, and (c) ^16^O_2_ and H_2_^18^O.

The GC-mass spectrum of thioanisole oxide, which exhibits an ion peak at *m*/*z* = 140, is shifted two mass unit higher to *m*/*z* = 142 (90% ^18^O labelled oxygen atoms) when the reaction is carried out with ^18^O_2_ (Fig. S17, ESI†). The labeling experiment clearly indicates the incorporation of one oxygen atom from molecular oxygen into the product. Mixed labeling experiments on thioanisole and styrene using ^16^O_2_ and H_2_^18^O reveal about 30% (using 25 equiv. of H_2_^18^O) and 55% (using 50 equiv. of H_2_^18^O) ^18^O atom incorporation from water into thioanisole oxide and styrene epoxide, respectively (Scheme 3, Fig. 4 and S17, ESI†). The reaction of complex **1** with styrene and ^18^O_2_ shows about 40% incorporation of one labelled oxygen atom into the epoxide product (Fig. 4). The low incorporation of labelled oxygen atoms from ^18^O_2_ indicates that water present in the solvent may exchange with the oxidant responsible for the epoxidation reaction. When the reaction of **1** with styrene is performed using ^16^O_2_ and different equivalents of H_2_^18^O, the percentage of labelled oxygen incorporation into the epoxide product increases with an increasing amount of H_2_^18^O (Fig. S18, ESI†). A maximum of 68% ^18^O incorporation into styrene epoxide takes place with 125 equivalents of H_2_^18^O, supporting the exchange of H_2_^18^O with the oxygen atom of the active oxidant. Iron(iv)-oxidants are electrophilic in nature and exchange their oxygen atoms with water.^34^–^36^ Although no iron–oxygen intermediate could be detected, circumstantial evidence through interception and mechanistic studies support the generation of iron(iv)–oxo species upon the oxidative decarboxylation of **1**.

**Scheme 4:**
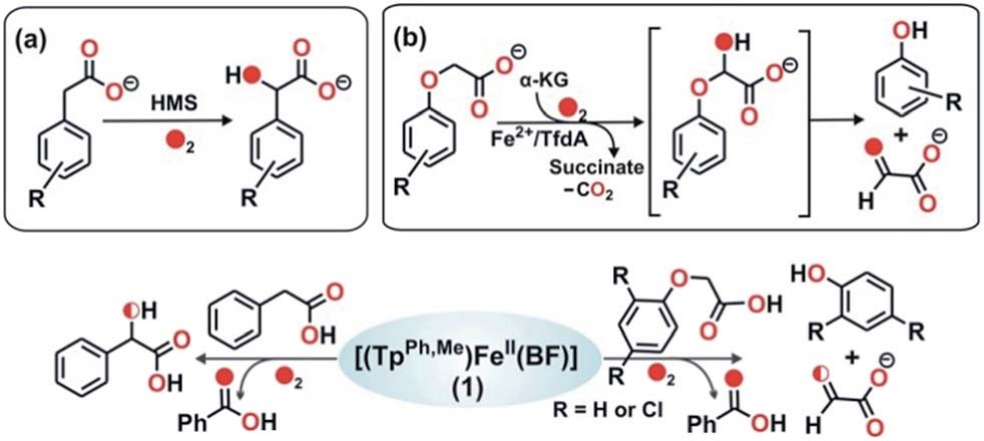
Reactions catalyzed by (a) HMS, and (b) TfdA enzyme. Bottom: reactions between **1** and phenylacetate, and between **1** and phenoxyacetate.

To further study the ability of the oxidant to perform various biologically relevant hydroxylation reactions, we have carried out a reaction between **1** and phenylacetic acid. Phenylacetate is a substrate in the bio-catalytic pathway of the α-ketoglutarate dependent enzyme, hydroxymandelate synthase (HMS) (Scheme 4a). In the case of HMS, an iron(iv)–oxo oxidant hydroxylates the benzylic carbon of the 4-hydroxyphenylacetate intermediate to form hydroxymandelate.^37^–^39^ Complex **1** reacts with phenylacetate (5 equiv.) to form about ∼70% mandelic acid (Scheme 4 and Fig. S19, ESI†). The GC-mass spectrum of the methyl ester of mandelic acid shows a molecular ion peak at *m*/*z* = 166, which shifts to 168 (65% ^18^O incorporation) upon reaction with ^18^O_2_ (Fig. S20, ESI†). When the same reaction is performed in the presence of H_2_^18^O (25 equiv.) and ^16^O_2_, about 30% ^18^O incorporation is estimated (Fig. S20c, ESI†). These results clearly indicate that the oxygen atom in mandelic acid is derived from a putative iron(iv)–oxo oxidant which exchanges its oxygen atom with water.

The reactivity of **1** towards other biologically important substrates such as phenoxyacetic acid and 2,4-dichlorophenoxyacetic acid (2,4-D) was also investigated. In the biodegradation pathway of 2,4-dichlorophenoxyacetic acid (2,4-D), an α-ketoglutarate-dependent enzyme, 2,4-dichlorophenoxyacetate dioxygenase encoded by the tfdA gene (TfdA), is involved in the first step.^40^,^41^ The TfdA enzyme hydroxylates the 2,4-D substrate to form an unstable hemiacetal intermediate that spontaneously degrades to 2,4-dichlorophenol and glyoxylate (Scheme 4b). During this process one oxygen atom is incorporated into the ether bond of 2,4-D.^42^

Complex **1** reacts with phenoxyacetic acid (5 equiv.) to form about 18% phenol (Scheme 4, Fig. S21 and S22, ESI†). Interestingly, when 2,4-dichlorophenoxyacetic acid is used as a substrate, 2,4-dichlorophenol (∼20%) is obtained (Scheme 4, Fig. S23 and S24, ESI†). The production of phenols from phenoxyacetic acids by complex **1** thus functionally mimics the reaction catalyzed by TfdA enzyme. The labeling experiment with ^16^O_2_ and H_2_^18^O shows no incorporation of labelled oxygen into the phenol product derived from phenoxyacetic acid, suggesting that iron(iv)=O derived from complex **1** hydroxylates the ether oxygen bond of phenoxyacetic acid which subsequently cleaves between the aliphatic side chain and the ether oxygen bond to form phenol.

**Scheme 5:**
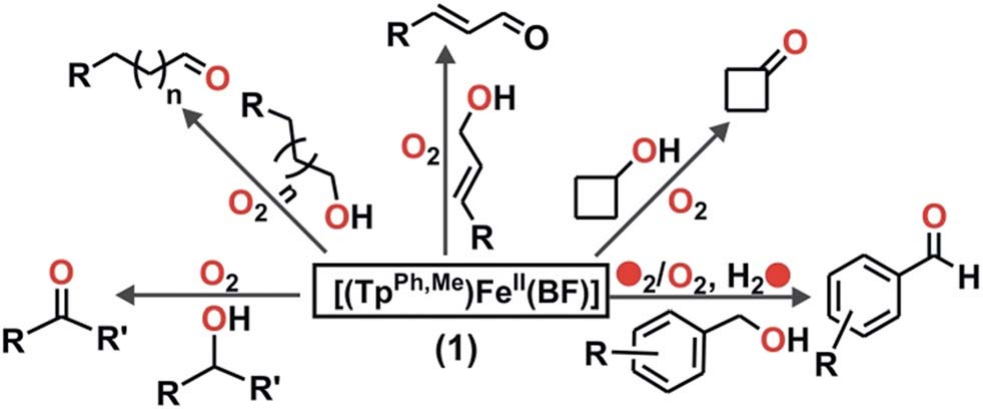
Products derived from different alcohols used as indirect probes to intercept the active iron–oxygen oxidant from **1**.

Intriguing results were obtained in the reactions of complex **1** with allylic and benzylic alcohols (Scheme 5). In the reaction with benzyl alcohol (20 equiv.), about 75% benzaldehyde is formed (Fig. S25, ESI†). While more than 95% interception is estimated for both *p*-nitrobenzyl alcohol and *p*-hydroxybenzyl alcohol, around 75% interception is found with 3-methoxybenzyl alcohol. In both cases, the corresponding aldehydes are obtained as oxidation products (Fig. S26–S28, ESI†). Phenethyl alcohol smoothly oxidizes to phenylacetaldehyde with 45% conversion (Fig. S29, ESI†). With the aliphatic primary alcohol 1-octanol, 65% 1-octanal is obtained (Fig. S30, ESI†). Cyclohexanol intercepts the active oxidant to an extent of 45% with cylohexanone as the only product (Fig. S31, ESI†). The secondary alcohol 1-phenyl ethanol (10 equiv.) is oxidized selectively to acetophenone in good yield (65%) (Fig. S32, ESI†). In all these reactions, no overoxidation is observed confirming the selectivity of the oxidation reaction by complex **1**. Cinnamaldehyde (10 equiv.), a substrate containing both C=C and –OH groups (Scheme 5 and Fig. S33, ESI†), exclusively forms cinnamaldehyde with no epoxide and almost quantitative (90%) interception of the oxidant takes place.

**Scheme 6:**
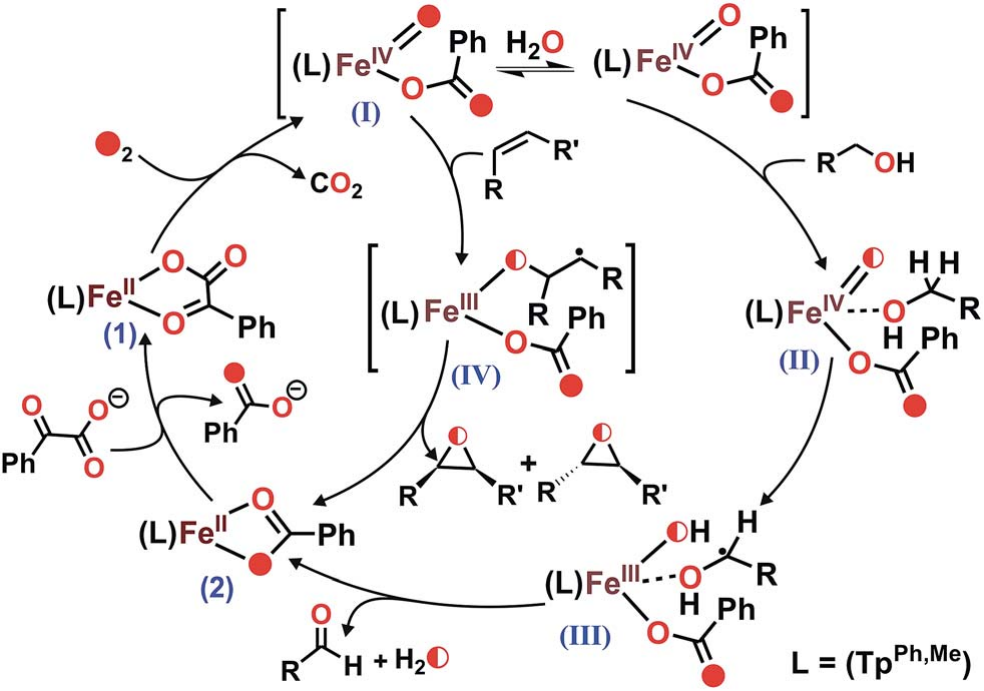
Proposed mechanisms for the formation of high-valent iron–oxo oxidant from **1** and the catalytic oxidation of substrates.

Heme and nonheme iron–oxo intermediates have been reported to oxidize alcohols exclusively by hydrogen atom abstraction from the α-CH of alcohol.^43^,^44^ High-valent iron–oxo complexes show very large (>10) KIE values. Intermolecular competitive oxidation of a mixture of PhCH_2_OH and PhCD_2_OH (90% D) by complex **1** gives a kinetic isotope effect (KIE) value of 2.33. A low KIE with a value of around 2.2, however, has been obtained in the manganese-catalyzed oxidation of alcohols,^45^–^47^ where high-valent Mn–oxo species have been proposed as active intermediates. Cyclic alcohols such as cyclobutanol or cyclopentanol are frequently used as mechanistic probes to distinguish between a one-electron (open chain aldehyde) *versus* two-electron (cyclic ketone) process in alcohol oxidation reactions.^48^ Complex **1**, upon reaction with cyclobutanol, afforded cyclobutanone as the exclusive product suggesting that alcohol oxidation by complex **1** takes place *via* a two-electron process (Scheme 5 and Fig. S34, ESI†). The excellent selectivity for alcohol oxidation along with the KIE value suggests that hydroxyl radical is not involved in the oxidation pathway, rather that a high-valent iron–oxo species (**I** in Scheme 6) is the active oxidant generated from **1**. Abstraction of the α-H of the metal bound substrate (**II**) results in a radical-based intermediate (**III**) which upon electron transfer rearranges to aldehyde and iron(ii)–benzoate species (**2**). The oxidation of 4-nitrobenzyl alcohol with ^18^O_2_ by complex **1** reveals no incorporation of labelled oxygen into 4-nitrobenzaldehyde. A mixed labeling experiment with ^16^O_2_ in H_2_^18^O also shows no incorporation of labelled oxygen into the aldehyde product (Fig. S35, ESI†). These results indicate that the oxygen atom in the aldehyde product does not derive from the oxo–iron(iv) unit. Therefore, the aldehyde product is formed by initial hydrogen atom abstraction followed by electron transfer reaction and not by a *gem*-diol pathway (Scheme 6).^48^ For the epoxidation of alkenes, isolation of both the *cis*-and *trans*-epoxide from *cis*-stilbene indicates that the reaction takes place *via* a step-wise process, not through a concerted pathway. Of note, for the iron(ii)–benzoylformate complex of Tp^Me2^ ligand retention of configuration in the epoxide product was reported.^27^ The proposed radical intermediate (**IV**), formed upon one electron transfer to the iron(iv)–oxo, may react with excess oxygen to give more epoxide (for cyclooctene)^49^ or allylic oxidation products (for cyclohexene). However, the product distribution does not change in the presence of radical quenchers such as methanol or TEMPO. These results indicate that the reaction does not proceed *via* a radical auto-oxidation pathway.

It has been reported that the iron(ii)–benzoylformate of Tp^Ph2^ ligand reacted with dioxygen to undergo oxidative decarboxylation concomitant with intra-ligand hydroxylation.^21^ On the contrary, complex **1** is quantitatively converted to **2**, but no intra-ligand hydroxylation is observed. In the absence of any substrate, the O_2_-derived oxidant from **1** likely decays *via* some unproductive pathway. But the CT bands of the iron(ii)–benzoylformate complex could be regenerated by addition of a solution of benzoylformic acid (HBF) and NEt_3_ (1 equiv.) (Fig. S36, ESI†) to the final oxidized solution after the reaction of **1** with O_2_ in the presence of excess substrate. This process could be repeated up to 4 cycles. This prompted us to investigate the catalytic reactivity of complex **1** toward alcohols, sulfides and alkenes (Table 1 and Experimental section). Acetonitrile was found to be the best solvent for catalytic studies. The reaction with 25 equiv. of NaBF and 50 equiv. of benzyl alcohol affords benzaldehyde with a turnover number (TON) of 14. 4-Nitrobenzyl alcohol is found to be the best substrate with a TON of 16 for the formation of 4-nitrobenzaldehyde and a TON of around 19 for the conversion of BF to benzoate. Almost 50% (TON of 5) of 4-nitrobenzyl alcohol could be selectively converted to 4-nitrobenzaldehyde using 10 equiv. each of substrate and BF. The efficiency of the catalyst was studied by using structurally diverse secondary and primary alcohols under the optimized reaction conditions. Almost similar TONs are obtained with cinnamyl alcohol as substrate (Table 1). Of note, a control experiment using Fe(ClO_4_)_2_·*x*H_2_O instead of complex **1** under similar experimental conditions shows no TON, emphasizing the role of iron(iv)=O generated from **1** in accomplishing catalytic transformations. The effectiveness of the catalyst in the oxidation of 4-nitrobenzyl alcohol was checked with increasing concentration of BF. The TON of aldehyde increases up to 16 with increasing concentration of BF (35 equiv.) (Fig. S37, ESI†). The addition of more BF/substrate did not improve the catalytic TON. In case of 4-hydroxybenzyl alcohol, the poor yield of aldehyde can be attributed to the coordination of phenolate oxygen to the iron(ii) center which prevents BF from coordinating to the iron center after the first cycle.

**Table 1:** Catalytic oxidation of substrates by **1** using dioxygen as the oxidant^*a*^

Entry	Substrate (S)	Product (S^ox^)	TON^*f*^ (BF)	TON^*e*^ (S^ox^) (%)
1^*c*^	Benzyl alcohol	Benzaldehyde	18 ± 0.5	14 ± 1 (28)
2^*b*^	4-Hydroxybenzyl alcohol	4-Hydroxy benzaldehyde	4 ± 0.5	2 ± 0.5 (4)
3^*b*^	4-Nitrobenzyl alcohol	4-Nitrobenzaldehyde	19 ± 0.5	16 ± 1 (32)
4^*d*^	4-Nitrobenzyl alcohol	4-Nitrobenzaldehyde	8 ± 1	5 ± 1 (50)
5^*b*^	3-Methoxy benzyl alcohol	3-Methoxy benzaldehyde	13 ± 1	8 ± 0.5 (16)
6^*b*^	Cinnamyl alcohol	Cinnamaldehyde	14 ± 0.5	12 ± 1 (24)
7^*b*^	Cyclohexanol	Cyclohexanone	10 ± 1	3 ± 0.5 (6)
8^*b*^	1-Phenylethanol	Acetophenone	13 ± 1	8 ± 0.5 (16)
9^*b*^	1-Octanol	Octanal	11 ± 0.5	6 ± 1 (12)
10^*b*^	Phenethyl alcohol	Phenylacetaldehyde	10 ± 1	4 ± 0.5 (8)
11^*b*^	Thioanisole	Thioanisole oxide	11 ± 1	3 ± 0.5 (6)
12^*b*^	4-Methoxy thioanisole	4-Methoxy thioanisole oxide	12 ± 1	3 ± 0.5 (6)
13^*b*^	4-Methyl thioanisole	4-Methyl thioanisole oxide	16 ± 0.5	6 ± 0.5 (12)
14^*g*^	Dimethyl sulfide	Dimethyl sulfoxide	15 ± 0.5	6 ± 0.5 (6)
15^*b*^	Dimethyl sulfoxide	Dimethyl sulfone	10 ± 0.5	3 ± 0.5 (6)
16^*g*^	Styrene	Styrene oxide	15 ± 1	6 ± 0.5 (6)
17^*g*^	Cyclooctene	Cyclooctene oxide	12 ± 0.5	3 ± 0.5 (3)
18^*h*^	Styrene	Styrene oxide	8 ± 0.5	4 ± 0.5 (8)
19^*h*^	4-Methyl thioanisole	4-Methyl thioanisole oxide	7 ± 0.5	3 ± 0.5 (6)

^*a*^Reaction conditions: catalytic reactions were carried out in dry acetonitrile for 8 h.

^*b*^0.02 mmol (0.0137 g) catalyst, 50 equiv. (1 mmol) of substrate, 35 equiv. (0.7 mmol) of HBF + NEt_3_.

^*c*^NaBF (25 equiv., 0.5 mmol) was used instead of HBF + NEt_3_.

^*d*^0.02 mmol catalyst, 10 equiv. (0.2 mmol) of substrate, 10 equiv. (0.2 mmol) of HBF + NEt_3_.

^*e*^TON (S^ox^) = mol of product formed/mol of catalyst used.

^*f*^TON (BF) = (mol of benzoic acid formed – mol of catalyst used)/mol of catalyst used.

^*g*^0.02 mmol catalyst, 100 equiv. (2 mmol) of substrate, 35 equiv. (0.7 mmol) of HBF + NEt_3_.

^*h*^0.02 mmol catalyst, 50 equiv. (1 mmol) of substrate, 10 equiv. (0.2 mmol) of HBF + NEt_3_. The values in the parenthesis indicate the percentage conversion of the substrates.

Complex **1** acts as a catalyst in OAT reactions for sulfide and alkene oxidations (Table 1). The highest TON is obtained in the epoxidation of styrene (TON = 6). Under similar experimental conditions, cyclooctene shows a TON of 3 whereas *cis*-2-heptene and 1-octene exhibit no catalytic TON. Similarly, TONs of more than 6 are obtained in the oxidations of DMS and 4-methyl thioanisole (Table 1 and Fig. S38, ESI†). It is important to mention here that iron(ii)–benzoate complex [(Tp^Ph,Me^)Fe^II^(OBz)] (**2**) does not show any oxidation of substrates (thioanisole/cyclooctene/benzyl alcohol/styrene) under similar experimental conditions.

**Fig. 5:**
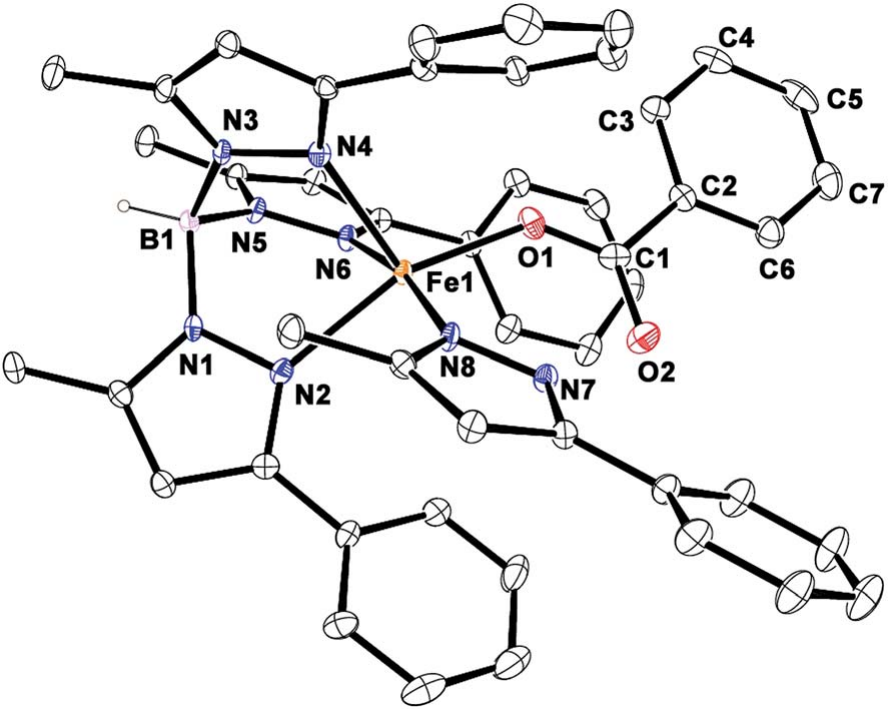
ORTEP plot of [(Tp^Ph,Me^)Fe^II^(OBz)(PzH)] (**2^PzH^**). All the hydrogen atoms except for B1 have been omitted for clarity. Selected bond lengths [Å] and angles [°] for **2^PzH^**: Fe1–N2 2.1412(13), Fe1–N4 2.1179(13), Fe1–N6 2.1788(13), Fe1–O1 2.0047(12), Fe1–N8 2.1430(13), N4–Fe1–O1 109.31(5), N4–Fe1–N2 94.42(5), N6–Fe1–N4 89.19(5), N4–Fe1–N8 96.22(5), N2–Fe1–O1 154.04(5), N8–Fe1–N6 168.97(5), N8–Fe1–O1 97.90(5), N6–Fe1–O1 89.30(5), N8–Fe1–N2 89.58(5), and N2–Fe1–N6 80.41(5).

Reactions with complex **1** and varying amounts of BF (1–5 equiv.) in the absence of an external substrate yield only one equivalent of benzoic acid. In none of the reactions is excess BF added to the reaction consumed. These observations rule out the possibility of a parallel path for consumption of BF in the catalytic process. In the presence of an external substrate only, BF is consumed further to generate the active oxidant in the catalytic cycle. However, low TONs are observed with substrates such as alkenes and sulfides compared to those for BF decarboxylation. The reason for the relatively low TONs for substrate oxidation with complex **1** was also investigated. The Tp^Ph,Me^ ligand has a tendency to hydrolyze resulting in the formation of free pyrazole (PzH) in solution. The free pyrazole can coordinate to the iron(ii)–benzoate center forming a stable complex. The ESI-MS of the solution after catalytic oxidation shows an ion peak at *m*/*z* = 697.1 with the isotope distribution pattern calculated for [(Tp^Ph,Me^)Fe(PzH)]^+^ (Fig. S39, ESI†). Moreover, X-ray quality single crystals of the 5-methyl-3-phenylpyrazole adduct of the iron(ii)–benzoate complex were isolated. The X-ray crystal structure (Fig. 5) of the complex [(Tp^Ph,Me^)Fe^II^(OBz)(PzH)] (**2^PzH^**) reveals a five-coordinate distorted square pyramidal geometry (*τ* = 0.25) at the iron center. Two nitrogen atoms N2 and N6 from the Tp^Ph,Me^ ligand, one carboxylate oxygen atom O1 from the mononuclear benzoate and a nitrogen atom N8 from the neutral pyrazole constitute the basal plane. The apical position is occupied by the N4 nitrogen from the Tp^Ph,Me^ ligand with an Fe1–N4 distance of 2.118(1) Å. The Fe–N(pyrazole) bond lengths are typical of high-spin iron(ii) complexes that have a Tp^Ph,Me^ ligand.^30^ The monodentate benzoate moiety is coordinated to the iron(ii) center with an Fe1–O1 distance of 2.005(1) Å (Table S2†). The iron nitrogen bond distances of the ligand (Fe1–N2 = 2.141(1) Å) and (Fe1–N6 = 2.179(1) Å) are distinctly elongated compared to the corresponding Fe–N(pyrazole) bonds of complex **1** creating a space to accommodate the extra pyrazole ligand. The carbonyl oxygen O2 of the benzoate moiety interacts with the hydrogen atom of the N7 nitrogen of neutral pyrazole through hydrogen bonding at a distance of 2.690 Å. The most weakly bound pyrazole nitrogen, N6, occupies *trans* to the neutral 5-methyl-3-phenylpyrazole with an N8–Fe1–N6 angle of 168.97(5)°. A similar structural motif has been observed in a related five-coordinate iron(ii) complex [Fe(Tp^Ph_2_^)(OAc)(3,5-Ph_2_pzH)].^21^ The hydrogen bonding interaction between the benzoate moiety and the pyrazole makes the complex **2^PzH^** more stable than **2**. Thus, accumulation of **2^PzH^** during the catalytic reaction results in the deactivation of the catalyst. Hydrolysis of one equivalent of the Tp^Ph,Me^ ligand can produce three equivalents of pyrazole, of which one equivalent gets coordinated to the iron(ii)–benzoate complex. Thus, the other two equivalents of pyrazole present in solution might quench the metal-based oxidant without oxidizing the substrate.

Several iron complexes have been reported to oxidize alcohols catalytically using oxidants such as hydrogen peroxides or peracids in the presence or absence of a co-catalyst.^50^–^54^ The aerobic oxidation of alcohols catalyzed by iron salts has also been documented in the literature.^55^,^56^ Nam and co-workers have reported the catalytic aerobic oxidation of substrates such as thioanisole and benzyl alcohol by a nonheme [(TMC)Fe^IV^(O)]^2+^ (TMC = 1,4,8,11-tetramethyl-1,4,8,11-tetraazacyclotetradecene) oxidant.^57^ Complex **1** represents a novel O_2_-dependent nonheme iron catalyst for the oxidation of alcohols, sulfides and alkenes.

## Conclusions

In summary, we have isolated and characterized a nonheme iron(ii)–benzoylformate complex of a facial tridentate ligand, Tp^Ph,Me^. The complex exhibits versatile reactivity toward substrates such as sulfides, alkenes, and alcohols. An iron(iv)–oxo species, generated *in situ* in the oxidative decarboxylation of coordinated benzoylformate, is postulated as the active oxidant. The iron(ii)–benzoylformate complex oxidatively converts phenylacetic acid to mandelic acid and phenoxyacetic acids to the corresponding phenols, mimicking the function of hydroxymandelate synthase (HMS) and 2,4-dichlorophenoxyacetate dioxygenase (TfdA), respectively. Furthermore, the complex performs catalytic oxidation of alcohols to aldehydes, and oxygen atom transfer reactions to olefins and sulfides using dioxygen as the oxidant.

## Experimental

All chemicals and reagents were purchased from commercial sources and were used without further purification. Solvents were distilled and dried before use. Preparation and handling of air-sensitive materials were carried out under an inert atmosphere in a glove box. Air-sensitive complexes were prepared and stored in an inert atmosphere. The ligand KTp^Ph,Me^ was synthesized according to the protocol reported in the literature.^58^

Fourier transform infrared spectroscopy on KBr pellets was performed on a Shimadzu FT-IR 8400S instrument. Elemental analyses were performed on a Perkin Elmer 2400 series II CHN analyzer. Electro-spray ionization mass spectra were recorded using a Waters QTOF Micro YA263. ^1^H NMR spectra were measured at room temperature using a Bruker DPX-500 spectrometer. Solution electronic spectra (single and time-dependent) were measured on an Agilent 8453 diode array spectrophotometer. GC-MS measurements were carried out using a Perkin Elmer Clarus 600 using an Elite 5 MS (30 m × 0.25 mm × 0.25 μm) column with a maximum temperature of 300 °C. Labeling experiments were carried out using ^18^O_2_ gas (99 atom%) or H_2_^18^O (98 atom%) purchased from Icon Services Inc., USA.

### Synthesis of metal complexes

#### [(Tp^Ph,Me^)Fe^II^(BF)] (**1**)

A suspension of FeCl_2_ (0.064 g, 0.5 mmol) and Tp^Ph,Me^ (0.260 g, 0.5 mmol) in 10 mL of dichloromethane was vigorously stirred for 20 min. To the mixture, a methanolic solution (1 mL) of sodium benzoylformate (0.086 g, 0.5 mmol) was added. The resulting violet solution was allowed to stir for 8 h and then dried. The residue obtained was dissolved in dichloromethane (10 mL) and filtered. The filtrate was further dried, re-dissolved in acetonitrile and filtered. The filtrate was then evaporated to dryness. To the residue, 0.5 mL of dichloromethane and 5 mL of hexane were added and then stirred for 1 h during which time a violet precipitate settled down. The violet solid was then collected by filtration. X-ray quality single-crystals were obtained by slow evaporation from the solution of the complex in dichloromethane and hexane mixture. Yield: 0.27 g (80%). Anal. cald for C_38_H_33_BFeN_6_O_3_ (688.36 g mol^–1^): C, 66.30; H, 4.83; N, 12.21. Found: C, 66.11; H, 4.74; N, 12.62%. ESI-MS (positive ion mode, CH_3_CN): *m*/*z* = 689.1 (5%) {[(Tp^Ph,Me^)Fe(BF)] + H}^+^, 711.1 (5%) {[(Tp^Ph,Me^)Fe(BF)] + Na}^+^, 539.02 (100%) [Fe(Tp^Ph,Me^)]^+^. IR (cm^–1^): 3456(br), 2958(w), 2925(w), 2545(w), 1682(m), 1623(s), 1596(s), 1544(w), 1504(w), 1415(w), 1367(w), 1234(s), 1178(m), 1066(m), 1031(w), 989(w), 765(m), 690(s), 648(w). UV-vis: *λ*_max_, nm (*ε*, M^–1^ cm^–1^): 537 (315), 580 (300). ^1^H NMR (500 MHz, CD_3_CN): *δ* 57.5, 26.5, 17.0, 13.3, 7.8, 7.21, 6.8, –10.7 ppm.

#### [(Tp^Ph,Me^)Fe^II^(OBz)] (**2**)

A suspension of FeCl_2_ (0.064 g, 0.5 mmol) and Tp^Ph,Me^ (0.260 g, 0.5 mmol) in 10 mL of dichloromethane was vigorously stirred for 20 minutes. To the resulting mixture a methanolic solution (1 mL) of sodium benzoate (0.072 g, 0.5 mmol) was added. The colorless solution was allowed to stir for 8 h and then dried. The residue obtained was dissolved in dichloromethane (10 mL) and filtered. The filtrate was then dried and washed twice with 5 mL of hexane to obtain a pale white solid. Anal. cald for C_37_H_33_BFeN_6_O_2_ (660.35 g mol^–1^): C, 67.30; H, 5.04; N, 12.73. Found: C, 66.98; H, 5.30; N, 12.35%. ESI-MS (positive ion mode, CH_3_CN): *m*/*z* (%) = 661.23 (10%) {[(Tp^Ph,Me^)Fe(OBz)] + H}^+^, 539.02 (100%) [(Tp^Ph,Me^)Fe]^+^. IR (cm^–1^): 3431(br), 3259(w), 2675(w), 2493(w), 1600(s), 1556(m), 1541(s), 1424(s), 1389(m), 1411(m), 1399(w), 1176(m), 1067(m), 1031(w), 975(w), 765(s), 696(s). ^1^H NMR (500 MHz, CD_3_CN): *δ* 55.7, 21.6, 46.5, 26.0, 15.1, 11.4, 9.1, 8.1, 7.4, 6.9, –10.1 ppm.

### Reactivity with dioxygen

A solution of the complex (0.02 mmol) in 10 mL of dioxygen saturated CH_3_CN was allowed to stir at room temperature. After the reaction, the solvent was removed under reduced pressure and the residue was treated with 10 mL of 3 M HCl. The organic products were extracted with diethyl ether and the organic phases were washed with brine solution. The combined organic part was dried over Na_2_SO_4_ and the solvent was removed to dryness. The organic products were analyzed by ^1^H NMR spectroscopy or by GC-MS. Quantification of benzoic acid was done by comparing the peak area associated with two *ortho* protons of benzoic acid (*δ* 8.09–8.11 ppm) with one proton (*δ* 6.612 ppm) of 2,4-di-*tert*-butylphenol used as an internal standard.

### Interception studies with external substrates

Complex **1** (0.02 mmol) was dissolved in 10 mL of dry acetonitrile. To the solution the desired amount of external substrate was added. The solution was then saturated with dioxygen by purging dioxygen for 5 min and the reaction was allowed to continue for 1.5 h at room temperature under a dioxygen atmosphere. The reaction solution was then dried and the residue was treated with 3.0 M HCl solution (10 mL). The organic products were extracted with diethyl ether (3 × 20 mL) and washed with brine solution (2 × 20 mL). The combined organic phase was dried over Na_2_SO_4_ and the solvent was removed under high vacuum. The organic products were then analyzed using ^1^H NMR spectroscopy or GC-MS. The reaction with adamantane was carried out in benzene instead of acetonitrile. All experiments were performed in triplicate and the average values are reported in each case.

The products were quantified by comparing the peak area associated with two *ortho*-protons of benzoic acid (*δ* 8.09–8.11 ppm) with the peak area for the protons of the oxidized substrate. In certain cases, where the two *ortho* protons of benzoic acid could not be integrated, 2,4-di-*tert*-butylphenol (0.02 mmol) was used as an internal standard.

Quantification of thioanisole oxide was done by comparing the peak area of the three protons –CH_3_ (*δ* 2.76 ppm) with two *ortho*-protons of benzoic acid. The products derived from dihydroanthracene (DHA), fluorene, and adamantane were analyzed by GC-MS and quantified using calibration curves obtained for authentic compounds. The aldehyde products were quantified by comparing the peak area associated with one proton of aldehyde hydrogen (*δ* 9.5–10.1 ppm) with either two *ortho* protons of benzoic acid or by one proton (*δ* 6.612 ppm) of 2,4-di-*tert*-butylphenol used as an internal standard. Phenol and 2,4-dichlorophenol were quantified by comparing the peak area associated with two protons of phenol (*δ* 6.82 ppm) and one proton of 2,4-dichlorophenol (*δ* 6.99 ppm) with two ortho protons of benzoic acid (*δ* 8.10 ppm).

### Interception with dimethyl sulfoxide and dimethyl sulfide

Complex **1** (0.02 mmol) was dissolved in dry acetonitrile (10 mL). To the solution dimethyl sulfoxide (10 equiv., 0.2 mmol) or dimethyl sulfide (100 equiv., 2 mmol) was added. Dry dioxygen gas was bubbled through the solution for 5 min and the solution was stirred at room temperature under an oxygen atmosphere for 1.5 h. The solvent was then removed from the reaction mixture and distilled benzene (2 mL) was added to dissolve the residue. A slight excess of sodium dithionite (0.04 mmol) was then added to the benzene solution followed by the addition of D_2_O (1 mL) and the resulting solution was stirred for 15 min. To the solution was added 1,10-phenanthroline monohydrate (0.06 mmol) and stirred for an additional 30 min. The D_2_O layer was then collected and analyzed by ^1^H NMR spectroscopy. ^1^H NMR data of dimethyl sulfone from dimethyl sulfoxide (D_2_O, 500 MHz): *δ* 3.17 (s, 6H) ppm. ^1^H NMR data of dimethyl sulfoxide from dimethyl sulfide (D_2_O, 500 MHz): *δ* 2.78 (s, 6H) ppm.

### Interception with alkenes, alkanes and alcohols

Complex **1** (0.02 mmol) was dissolved in 2 mL of dioxygen saturated dry acetonitrile. To the solution external reagents, 100 equivalents (2 mmol) of alkenes or 10 equivalents of alcohols (0.2 mmol), were added. Dioxygen was purged through the solution for 5 min and the reaction solution was allowed to stir at room temperature for 1.5 h. The solution was then passed through a 15 cm silica column (60–120 mesh size) using dichloromethane/diethyl ether as the eluent. The combined organic phase was then analyzed by GC-mass spectrometry. The reaction with ethylbenzene was performed in benzene instead of acetonitrile. Quantification of the oxidized products was carried out by GC-mass spectrometry using standard calibration curves obtained with authentic compounds and naphthalene as an internal standard.

### Kinetic isotope effect

Complex **1** (0.02 mmol) was dissolved in 2 mL of acetonitrile. To the solution was added a 1 : 1 mixture of PhCD_2_OH (0.2 mmol, 90% D) and PhCH_2_OH (0.2 mmol). Dioxygen gas was then purged through the solution for 5 min and the reaction solution was allowed to stir at room temperature for 1.5 h. The solution was then passed through a 15 cm silica column (60–120 mesh size) using dichloromethane/diethyl ether as the eluent. The combined organic phase was then analyzed by GC-mass spectrometry. The percentage of benzaldehyde PhCHO/PhCDO formed was calculated using the relative intensities of the mass peaks in GC-MS.

### Catalytic experiments

The catalytic experiments were carried out using 0.02 mmol of the complex either in benzene or in acetonitrile under the different conditions mentioned in Table 1. The reactions were carried out for about 8 h.

#### NMR data of the oxidized products

Thioanisole oxide: ^1^H NMR *δ* 7.66 (m, 2H), 7.52 (m, 3H), 2.74 (s, 3H). Benzoic acid: ^1^H NMR (500 MHz, CDCl_3_) *δ* 8.11 (d, 2H), 7.63 (t, 1H), 7.48 (t, 2H). Benzaldehyde: ^1^H NMR (500 MHz, CDCl_3_) *δ* 10.02 (s, 1H), 7.53 (t, 2H), 7.63 (t, 1H), 7.88 (d, 2H). Dimethyl sulfoxide: ^1^H NMR (500 MHz, D_2_O) *δ* 2.78 (s, 6H). Dimethyl sulfone: ^1^H NMR (500 MHz, D_2_O) *δ* 3.17 (s, 6H). 4-Nitrobenzaldehyde: ^1^H NMR (500 MHz, CDCl_3_) *δ* 8.40 (d, 2H), 8.05 (d, 2H), 10.15 (s, 1H). 4-Hydroxybenzaldehyde: ^1^H NMR (500 MHz, CDCl_3_) *δ* 7.82 (d, 2H), 6.93 (d, 2H), 9.87 (s, 1H). Acetophenone: ^1^H NMR (500 MHz, CDCl_3_) *δ* 7.92 (d, 2H), 7.51 (m, 1H), 7.45 (d, 2H), 2.61 (s, 3H). Cinnamaldehyde: ^1^H NMR (500 MHz, CDCl_3_) *δ* 9.96 (d, 1H), 6.70–6.73 (m, 2H), 7.55–7.49 (aromatic protons 5H). 3-Methoxybenzaldehyde: ^1^H NMR (500 MHz, CDCl_3_) *δ* 7.48 (d, 2H), 7.42 (d, 1H), 7.22 (d, 2H), 9.96 (s, 1H). Octanal: ^1^H NMR (500 MHz, CDCl_3_) *δ* 0.88–2.35 (aliphatic chain protons) 9.81 (s, 1H). Phenol: *δ* 7.25 (t, 2H), 6.92 (t, 1H), 6.82 (d, 2H). 2,4-Dichlorophenol: *δ* 7.42 (s, 1H), 7.20 (d, 1H), 6.99 (d, 1H). Mandelic acid: *δ* 7.22–7.44 (m, 5H), 5.24 (s, 1H).

### X-ray crystallographic data collection, refinement and solution of the structures

X-ray single-crystal data for **1** and **2^PzH^** were collected at 120 K using Mo K_α_ (*λ* = 0.7107 Å) radiation on a SMART-APEX diffractometer equipped with CCD area detector. Data collection, data reduction, structure solution and refinement were carried out using the APEX II software package.^59^ The structure was solved by a direct method and subsequent Fourier analyses and refined by the full-matrix least-squares method based on *F*^2^ with all observed reflections.^60^ The non-hydrogen atoms were treated anisotropically. Routine SQUEEZE^61^ was applied to the intensity data of complex **2^PzH^** to take into account disordered solvent molecules.

Crystal data of **1**: MF = C_38_H_33_BFeN_6_O_3_, *M*_r_ = 688.36, orthorhombic, space group *Pbca*, *a* = 17.5030(17), *b* = 10.5880(10), *c* = 36.844(3) Å, *α* = 90.00°, *β* = 90.00°, *γ* = 90.00°, *V* = 6828.0(11) Å^3^, *Z* = 8, *ρ* = 1.339 mg m^–3^, *μ* Mo-Kα = 0.488 mm^–1^, *F*(000) = 2864, GOF = 1.045, a total of 68 324 reflections were collected in the range 1.60 ≤ *θ* ≤ 26.89, 7326 of which were unique (*R*_int_ = 0.0561). *R*_1_(*wR*_2_) = 0.0472(0.1576) for 449 parameters and 5408 reflections (*I* > 2*σ*(*I*)).

Crystal data of **2^PzH^**: MF = C_47_H_42_BFeN_8_O_2_, *M*_r_ = 817.55, triclinic, space group *P*1[combining macron], *a* = 12.6392(6), *b* = 12.6903(6), *c* = 16.0016(7) Å, *α* = 67.2510(10)°, *β* = 75.2650(10)°, *γ* = 79.5910(10)°, *V* = 2279.31(18) Å^3^, *Z* = 2, *ρ* = 1.191 mg m^–3^, *μ* Mo-Kα = 0.376 mm^–1^, *F*(000) = 854, GOF = 1.110, a total of 24 552 reflections were collected in the range 1.409 ≤ *θ* ≤ 27.49, 9704 of which were unique (*R*_int_ = 0.0176). *R*_1_(*wR*_2_) = 0.0378(0.1211) for 536 parameters and 8456 reflections (*I* > 2*σ*(*I*)).

## Supplementary Material

Supplementary information

Crystal structure data
