# Supplementary material for: Aerobic alcohol oxidation and oxygen atom transfer reactions catalyzed by a nonheme iron(ii)–α-keto acid complex
Source: Chem Sci. 2016 Apr 25;7(8):5322–31. doi: 10.1039/c6sc01476c (PMC6020522; doi:10.1039/c6sc01476c)
Supplement: Supplementary file 1 [file SC-007-C6SC01476C-s001.pdf]

Electronic Supplementary Material (ESI) for Chemical Science.
This journal is © The Royal Society of Chemistry 2016

Electronic Supplementary Information

for

Aerobic Alcohol Oxidation and Oxygen Atom Transfer Reactions Catalyzed by a Nonheme Iron(II)-α-Keto Acid Complex

Debobrata Sheet and Tapan Kanti Paine*

Department of Inorganic Chemistry, Indian Association for the Cultivation of Science,
2A & 2B Raja S. C. Mullick Road, Jadavpur, Kolkata-700032, India.
E-mail: ictkp@iacs.res.in

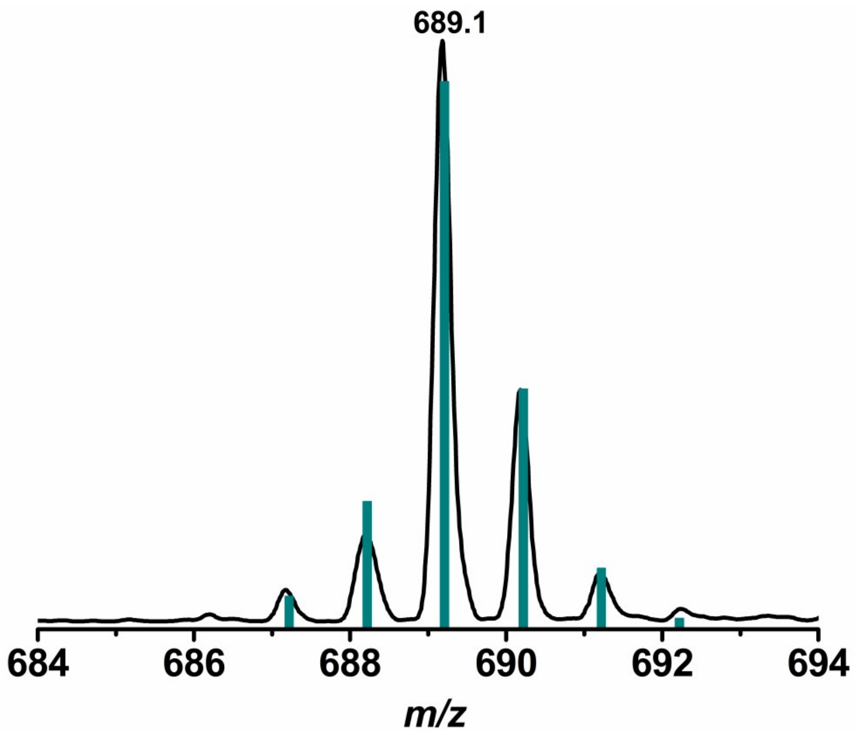

Fig. S1 ESI-mass spectrum (positive ion mode in CH_3CN) of [(Tp^{Ph,Me})Fe^{II}(BF)] (**1**). The bars indicate the simulated isotope distribution pattern of the complex.

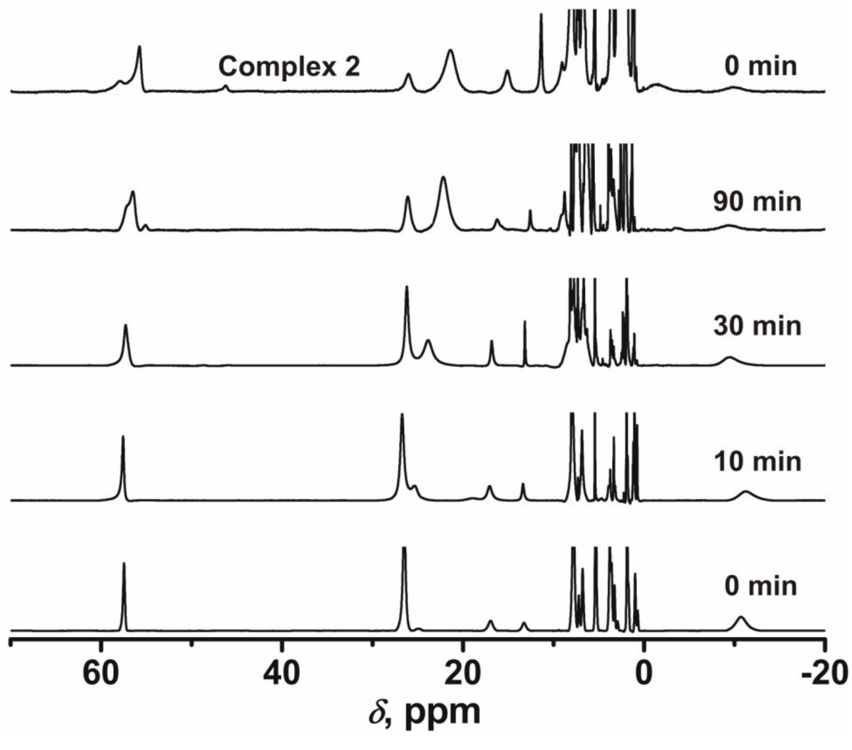

Fig. S2 Time-dependent ^{1}H NMR (500 MHz, CD_3CN, 295 K) spectra during the reaction of **1** with dioxygen. Top: ^{1}H NMR spectrum of [(Tp^{Ph,Me})Fe^{II}(OBz)] (**2**).

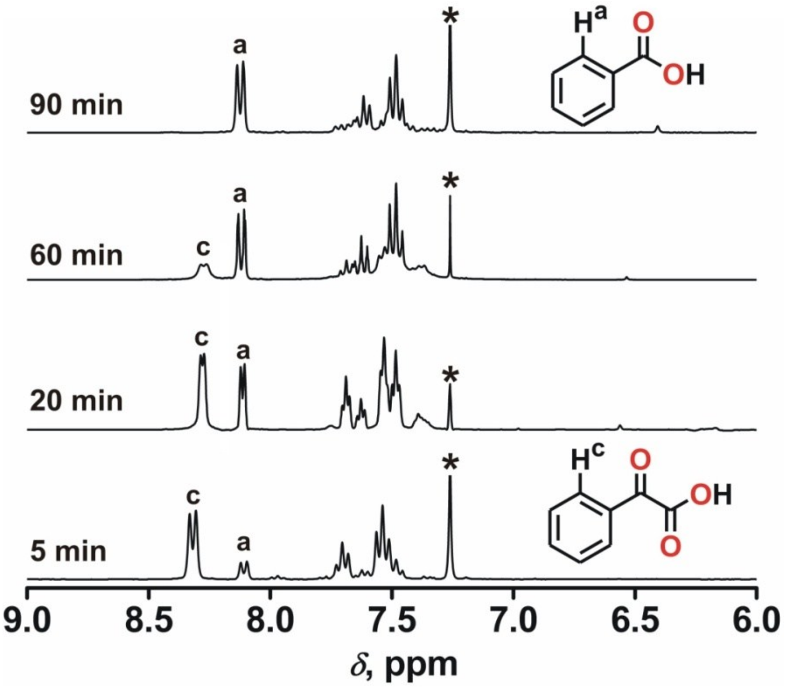

Fig. S3 Time-dependent ^{1}H NMR (500 MHz in $CDCl_3$ at 295 K) spectra of the organic products formed in the reaction of **1** with dioxygen. The peak marked with asterisk (*) is from residual solvent.

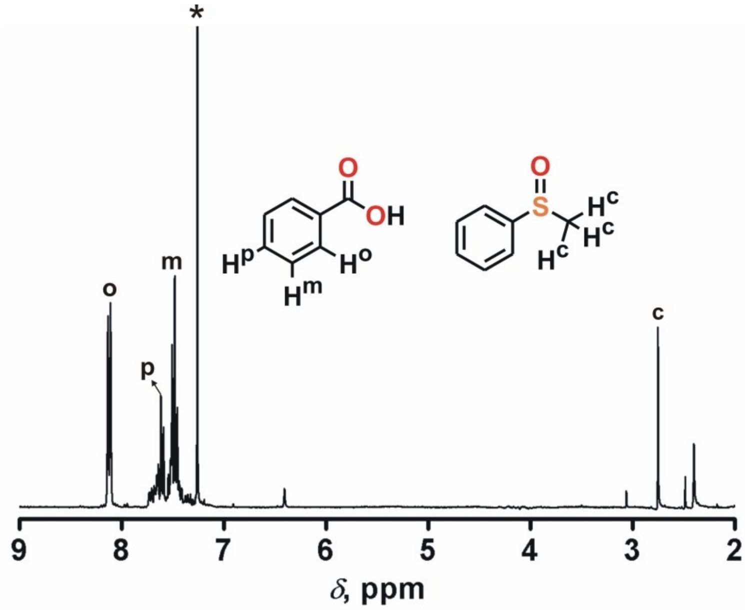

Fig. S4 ^{1}H NMR spectrum (500 MHz, $CDCl_3$, 295 K) of thioanisole-derived products obtained in the reaction of **1** with O_2 in the presence of thioanisole (10 equiv) in acetonitrile. The peak marked with asterisk (*) is from residual solvent.

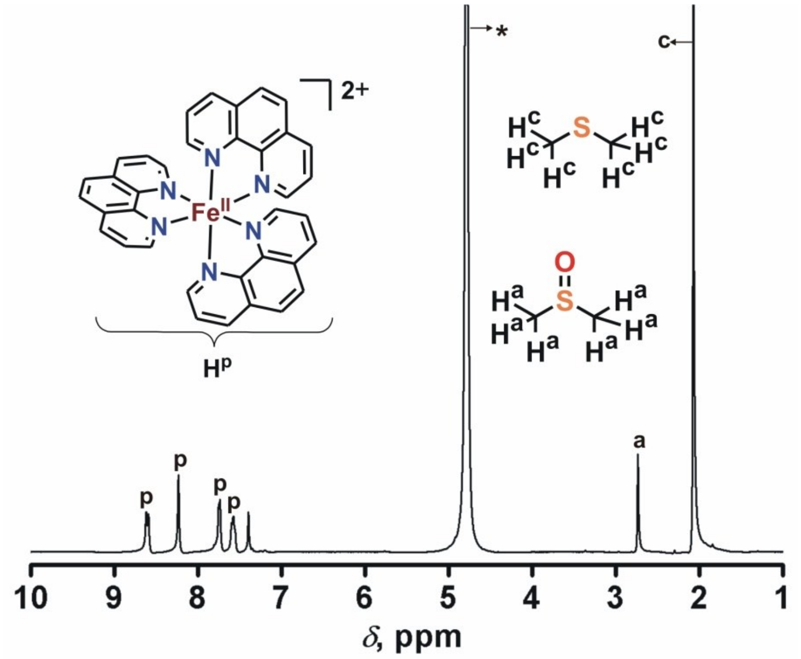

Fig. S5 ^{1}H NMR (500 MHz, D_2O, 295 K) spectrum of dimethyl sulfoxide formed in the reaction of **1** with O_2 in the presence of dimethyl sulfide (10 equiv) in acetonitrile. Peaks marked with (p) are from tris(1,10-phenanthroline)iron(II) complex and the peak marked with asterisk (*) is from residual solvent.

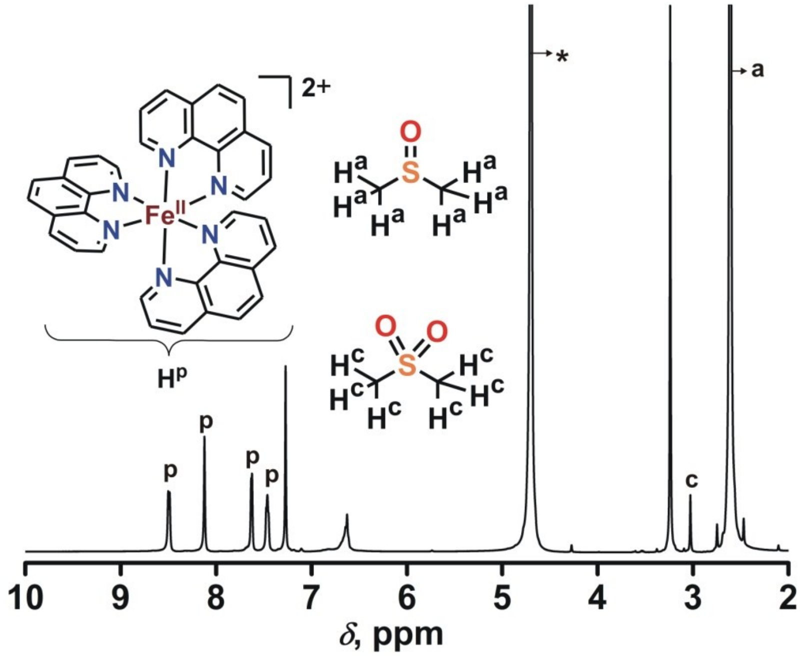

Fig. S6 ^{1}H NMR (500 MHz, D_2O, 295 K) spectrum of dimethyl sulfone formed in the reaction of **1** with O_2 in the presence of dimethyl sulfoxide (10 equiv) in acetonitrile. Peaks marked with (p) are from tris(1,10-phenanthroline)iron(II) complex and the peak marked with asterisk (*) is from residual solvent.

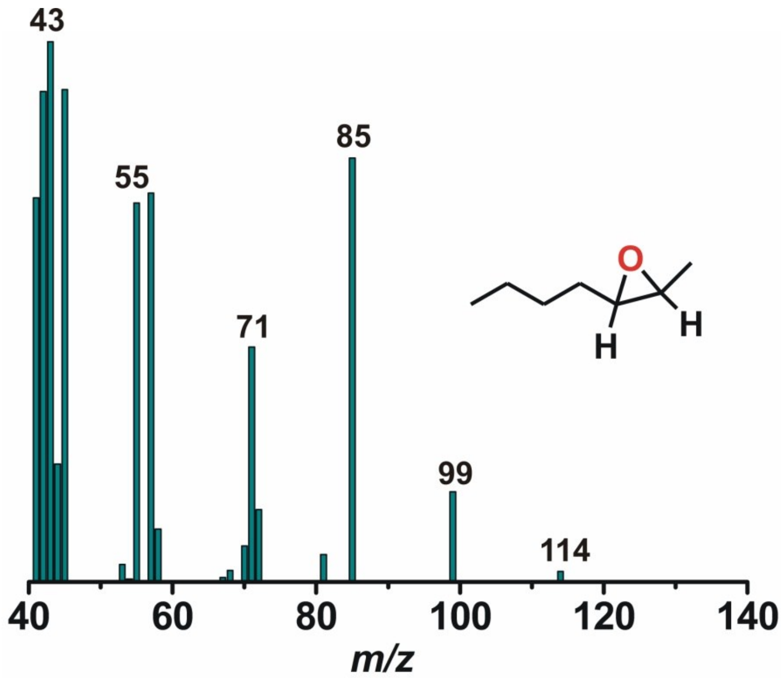

Fig. S7 GC-mass spectrum of *cis*-2-heptene oxide formed in the reaction of **1** with *cis*-2-heptene.

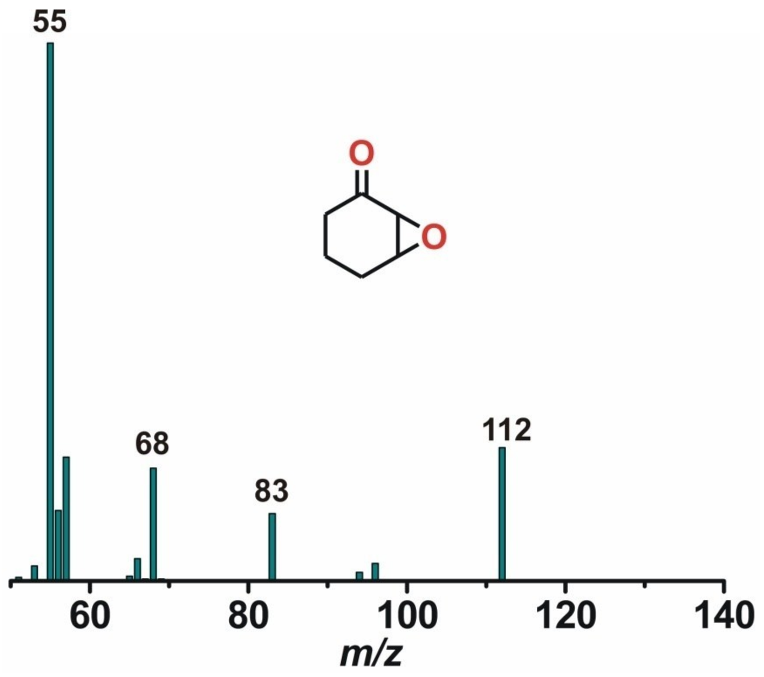

Fig. S8 GC-mass spectrum of 2-cyclohexenone oxide formed in the reaction of **1** with 2-cyclohexenone in acetonitrile.

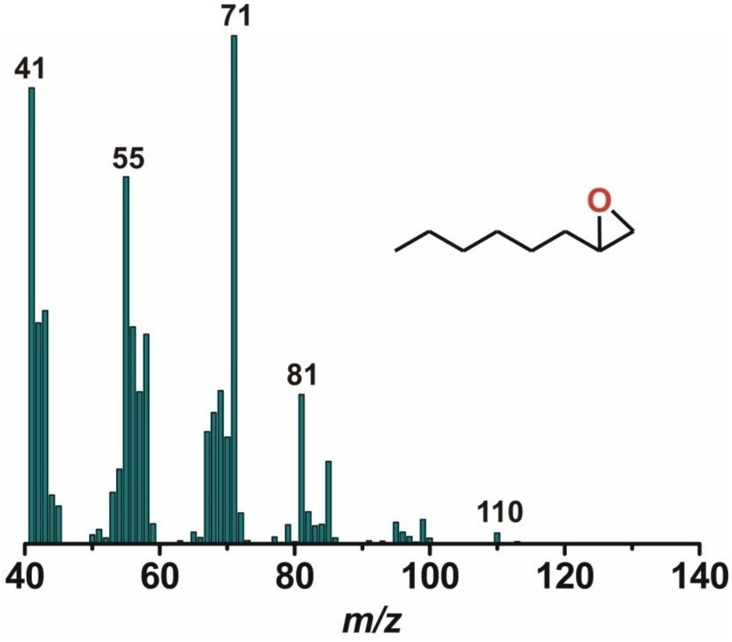

Fig. S9 GC-mass spectrum of 1-epoxyoctane formed in the reaction of **1** with 1-octene.

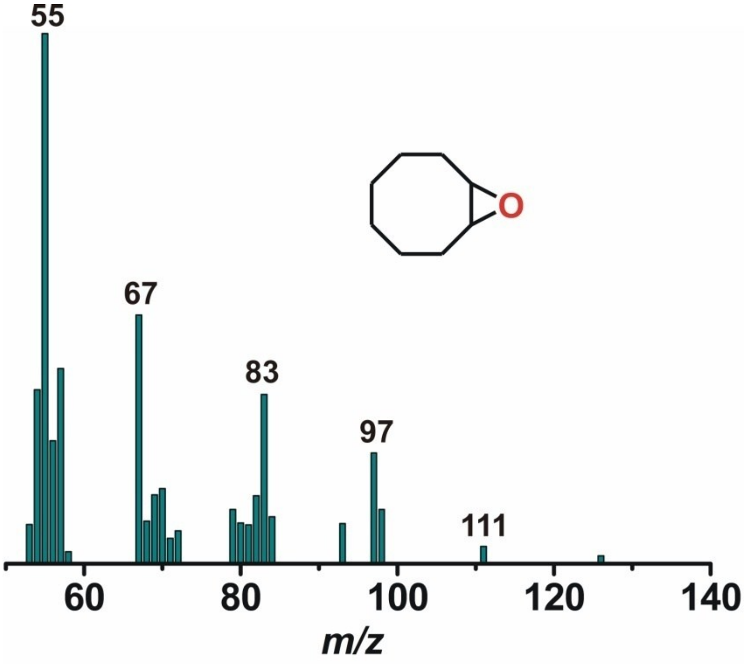

Fig. S10 GC-mass spectrum of cyclooctene oxide formed in the reaction between **1** and cyclooctene.

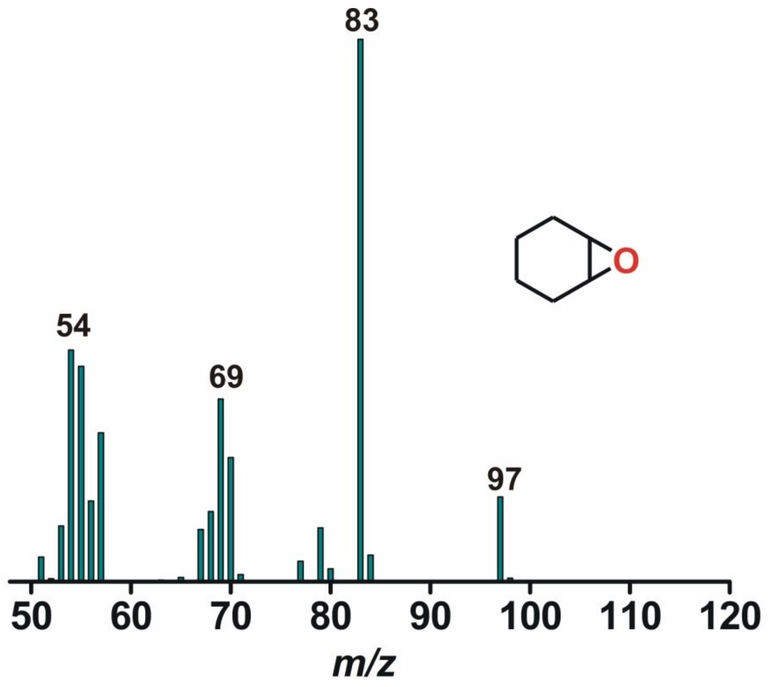

Fig. S11 GC-mass spectrum of cyclohexene oxide formed in the reaction of **1** with cyclohexene.

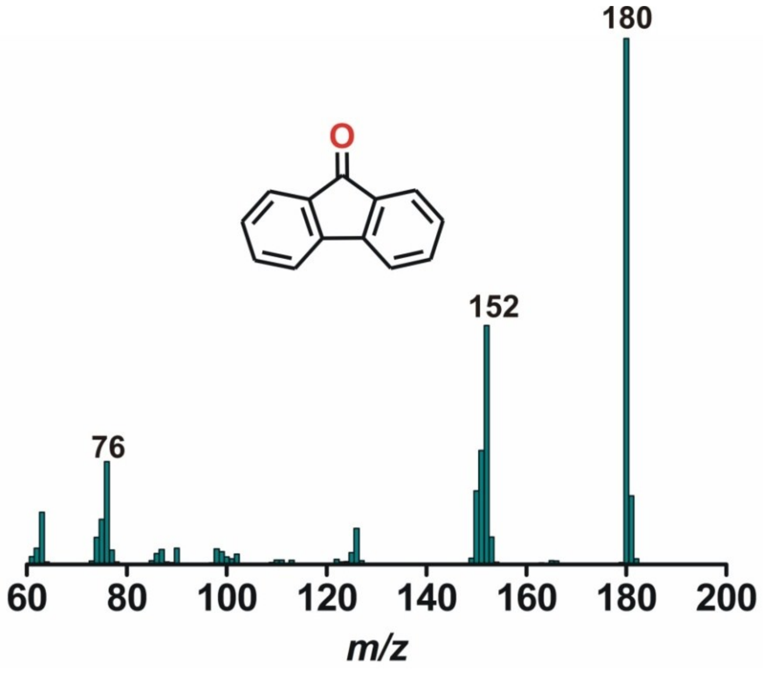

Fig. S12 GC-mass spectrum showing the formation of fluorenone in the reaction between **1** and fluorene in acetonitrile.

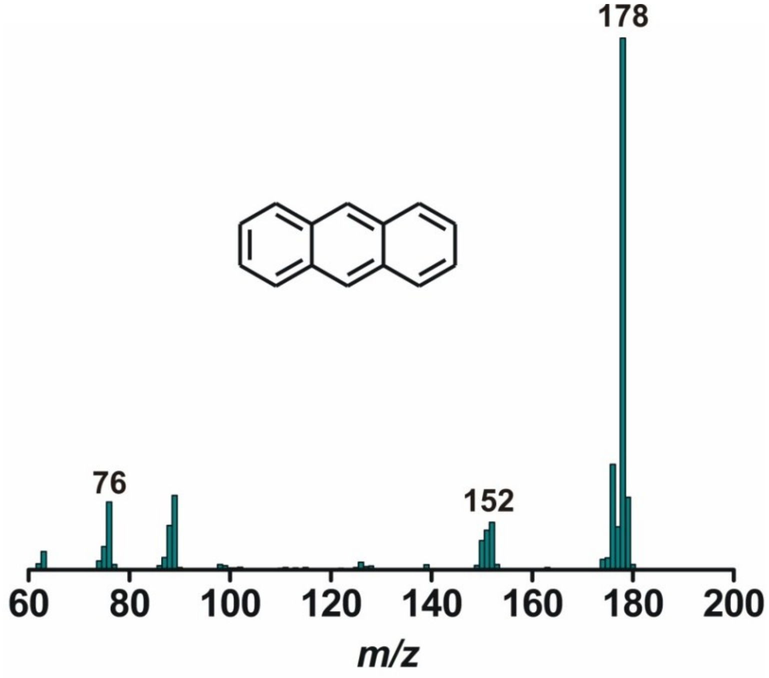

Fig. S13 GC-mass spectrum of anthracene formed in the reaction of **1** with 9,10-dihydroanthracene in acetonitrile.

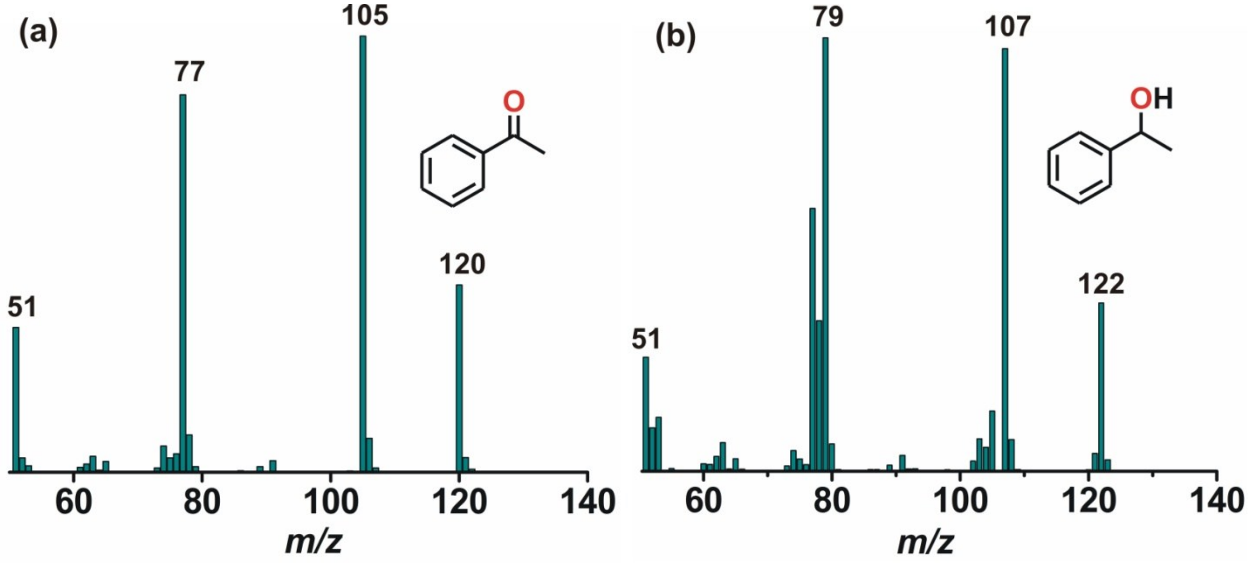

Fig. S14 GC-mass spectra of (a) acetophenone and (b) 1-phenylethanol formed in the reaction of **1** with ethylbenzene (100 equiv) in benzene.

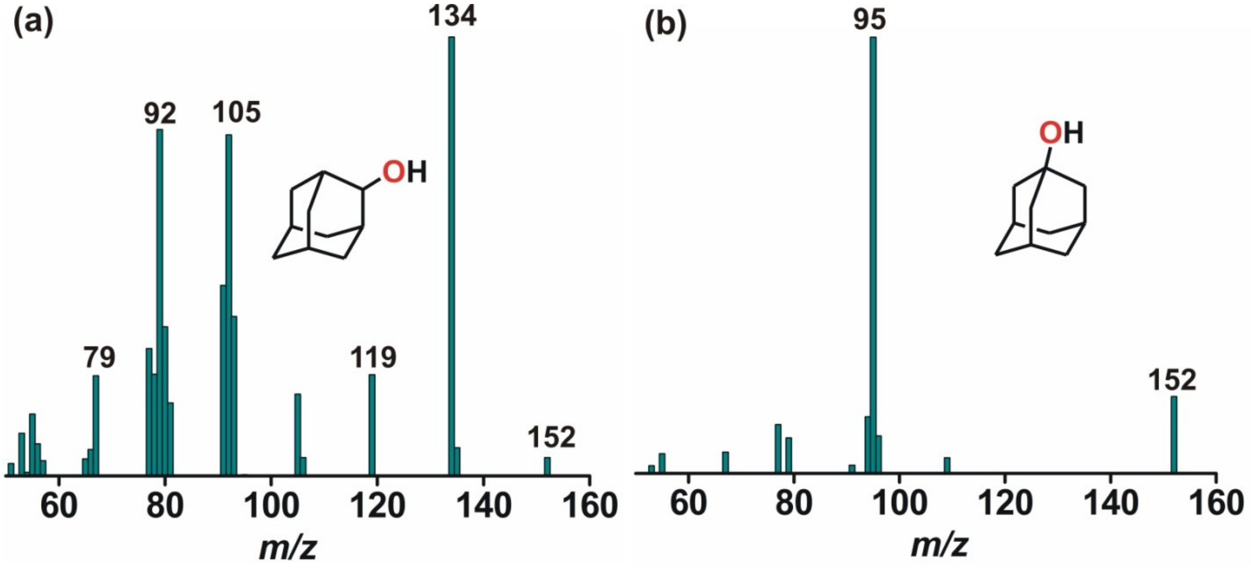

Fig. S15 GC-mass spectra of (a) 2-adamantanol and (b) 1-adamantanol formed in the reaction of **1** with adamantane (10 equiv) in benzene.

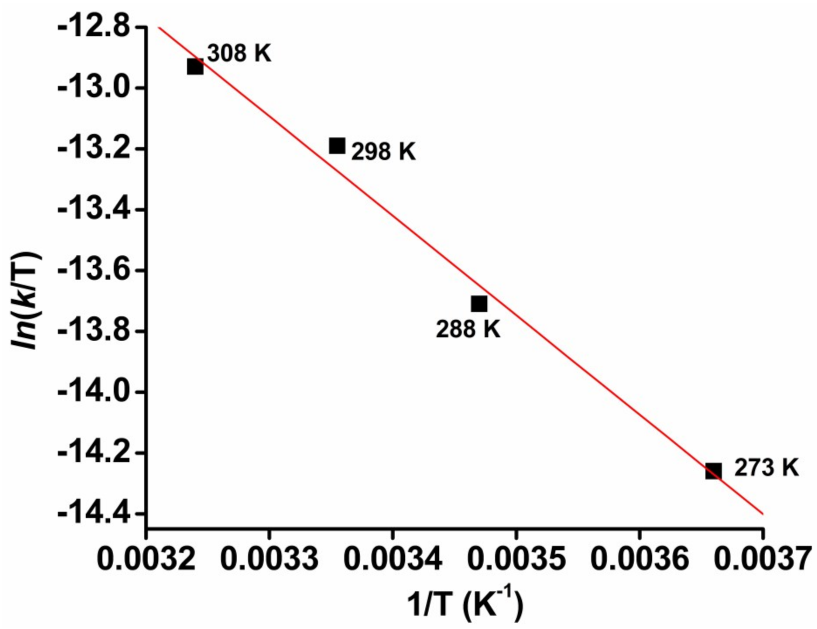

Fig. S16 Eyring plot for the reaction of **1** with O_2 in acetonitrile.

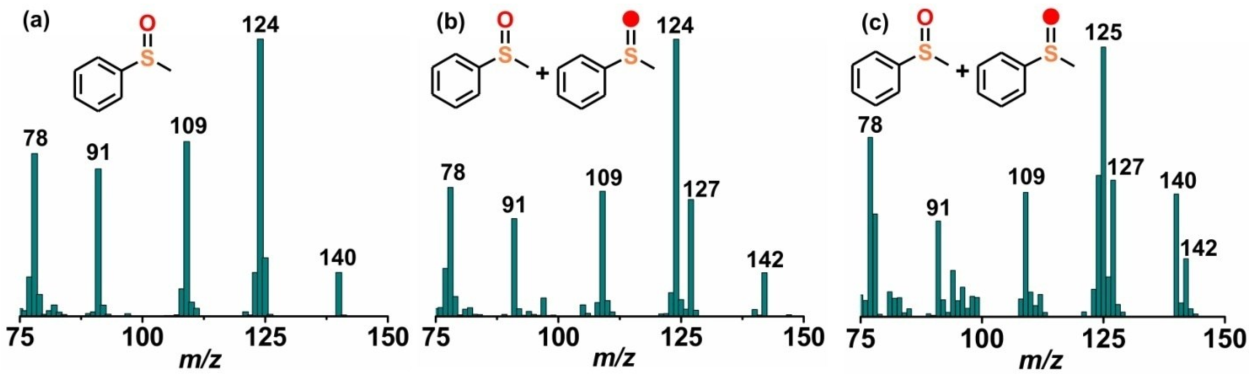

Fig. S17 GC-mass spectra of thioanisole oxide formed in the reaction of **1** with thioanisole in the presence of (a) $^{16}O_2$, (b) $^{18}O_2$, and (c) $^{16}O_2$ and $H_2{}^{18}O$.

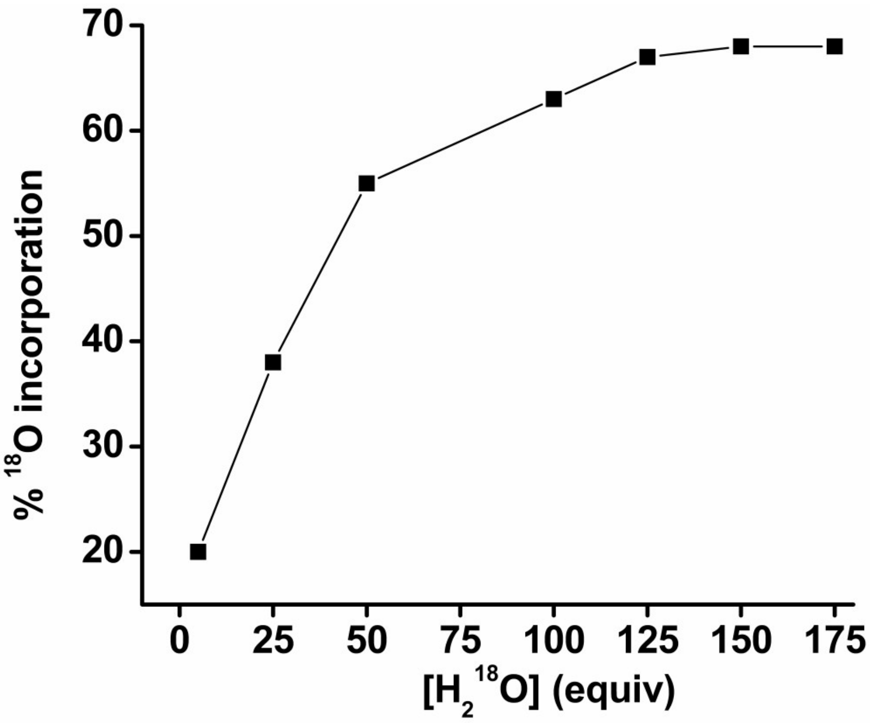

Fig. S18 Percentage of labeled oxygen into styrene oxide obtained in the oxidation of styrene by complex **1** and O_2 as a function of the concentration of $H_2{}^{18}O$.

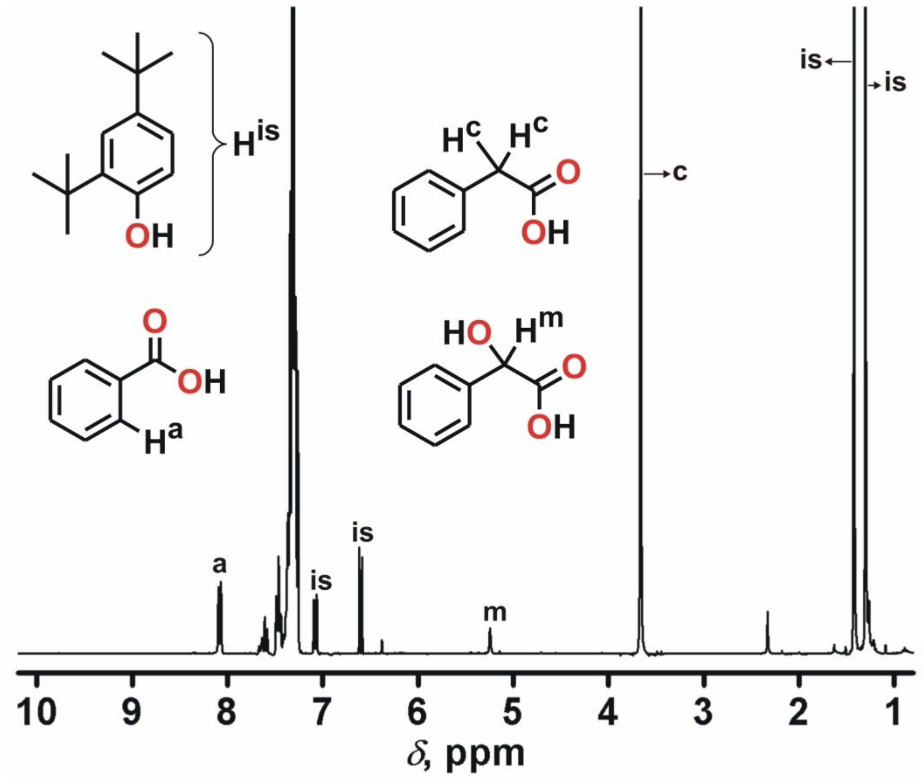

Fig. S19 ^{1}H NMR spectrum (500 MHz, $CDCl_3$, 295 K) of organic products derived from the reaction of **1** with phenylacetate (5 equiv) in acetonitrile.

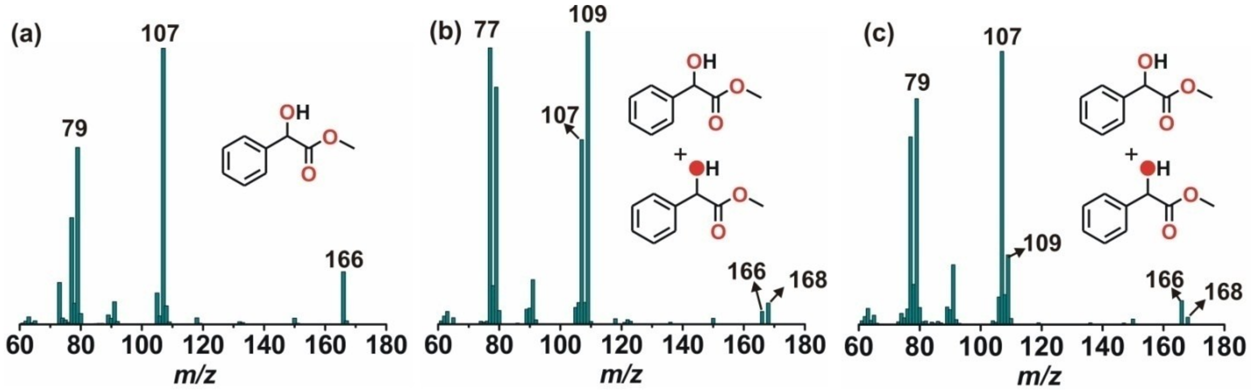

Fig. S20 GC-mass spectra of the (ester of) mandelic acid formed in the reaction of **1** with phenylacetic acid in acetonitrile in the presence of (a) $^{16}O_2$, (b) $^{18}O_2$, and (c) $^{16}O_2$ and $H_2{}^{18}O$.

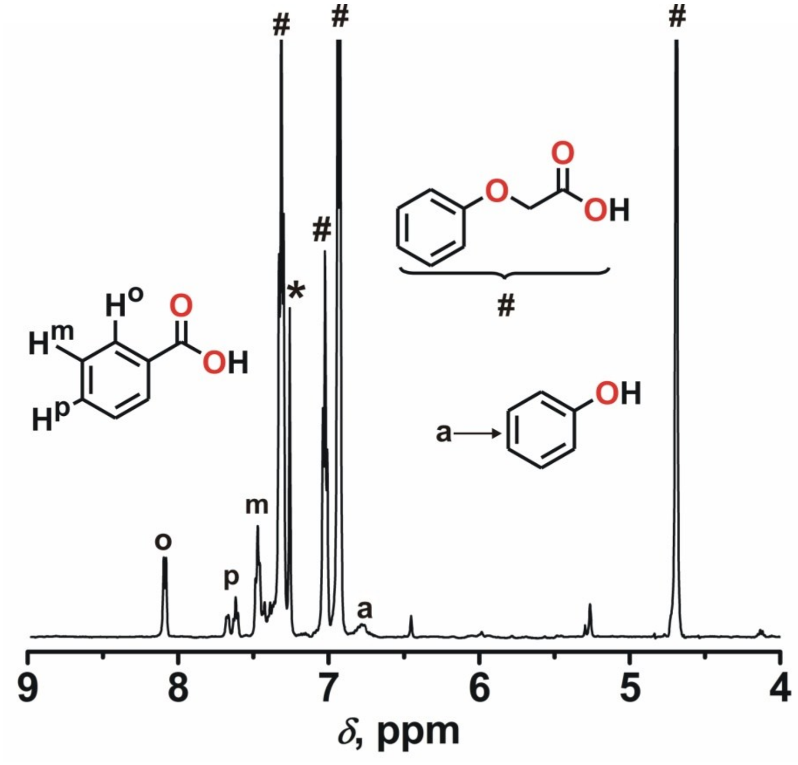

Fig. S21 ^{1}H NMR spectrum (500 MHz, $CDCl_3$, 295 K) of organic products derived from the reaction of **1** with phenoxyacetic acid (5 equiv) in acetonitrile.

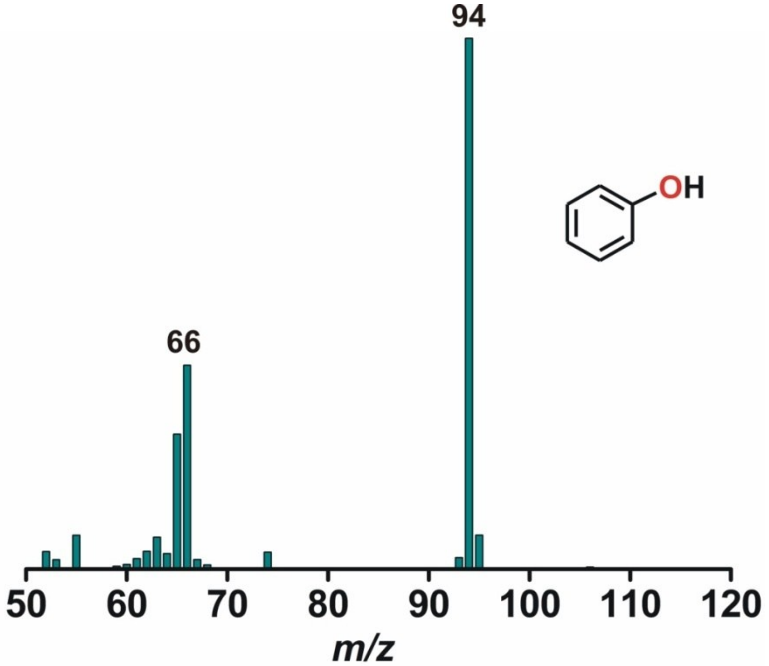

Fig. S22 GC-mass spectrum of phenol formed in the reaction of **1** with phenoxyacetic acid in acetonitrile.

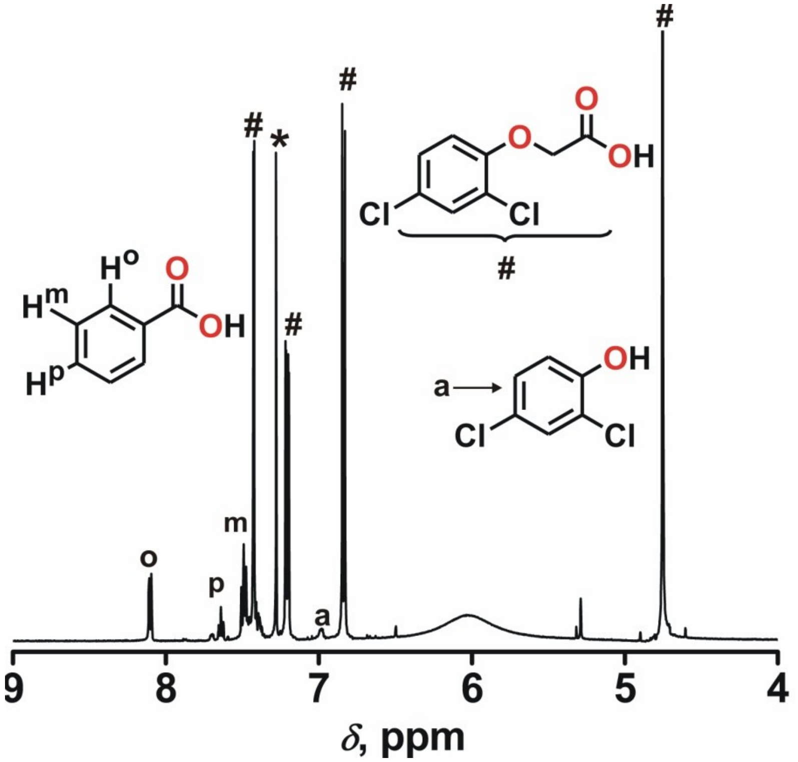

Fig. S23 ^{1}H NMR spectrum (500 MHz, $CDCl_3$, 295 K) of organic products from the reaction of **1** with 2,4 dichlorophenoxyacetic acid (5 equiv) in acetonitrile.

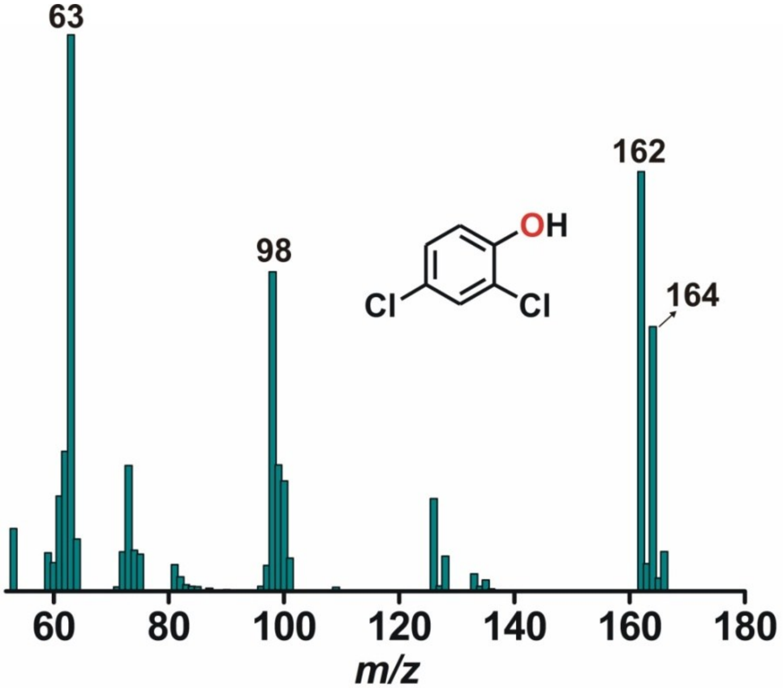

Fig. S24 GC-mass spectrum of 2,4-dichlorophenol formed in the reaction between **1** and 2,4-dichlorophenoxyacetic acid in acetonitrile.

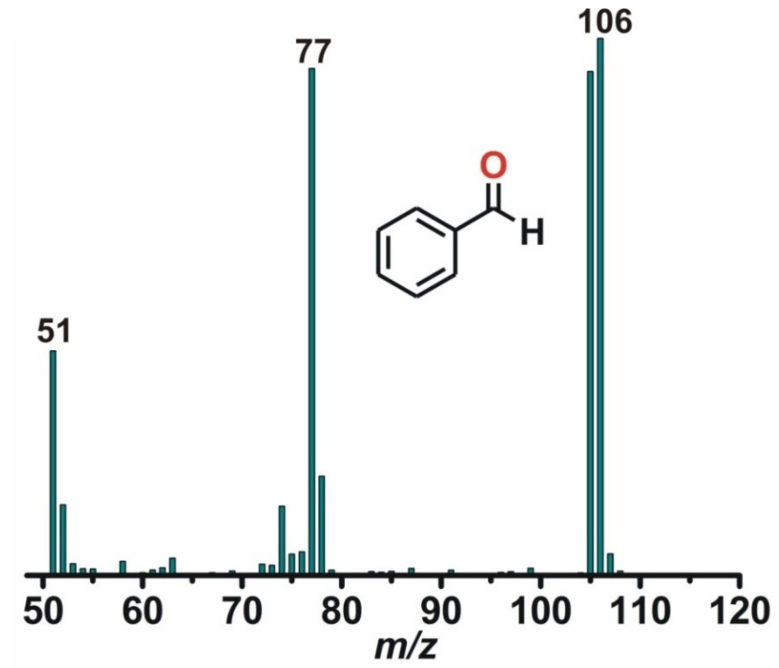

Fig. S25 GC-mass spectrum of benzaldehyde formed in the reaction between **1** and benzyl alcohol.

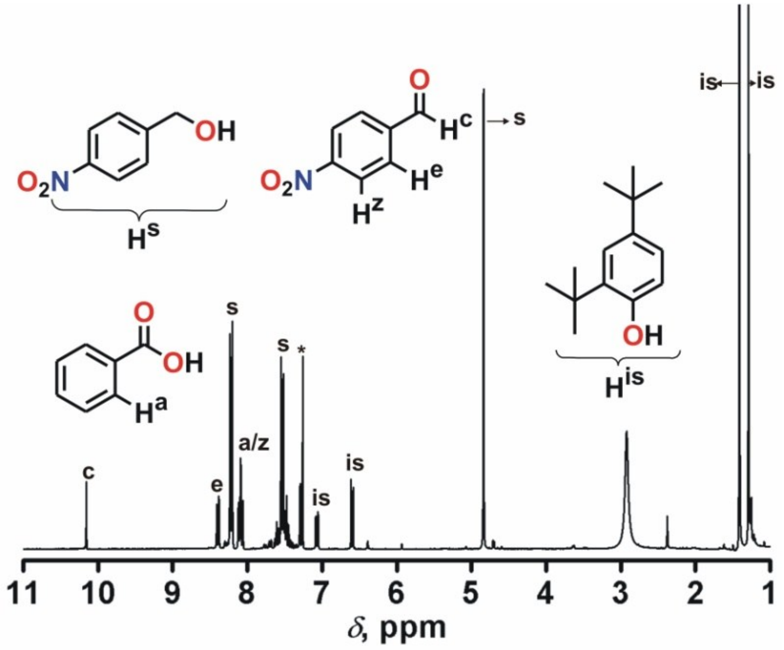

Fig. S26 ^1H NMR (500 MHz, $CDCl_3$, 295 K) spectrum of 4-nitrobenzaldehyde formed in the reaction of **1** with O_2 in the presence of 4-nitrobenzyl alcohol (10 equiv) in acetonitrile. Peaks marked with (is) are derived from 2,4-di-*tert*-butylphenol used as an internal standard and the peak with asterisk (*) is from residual solvent.

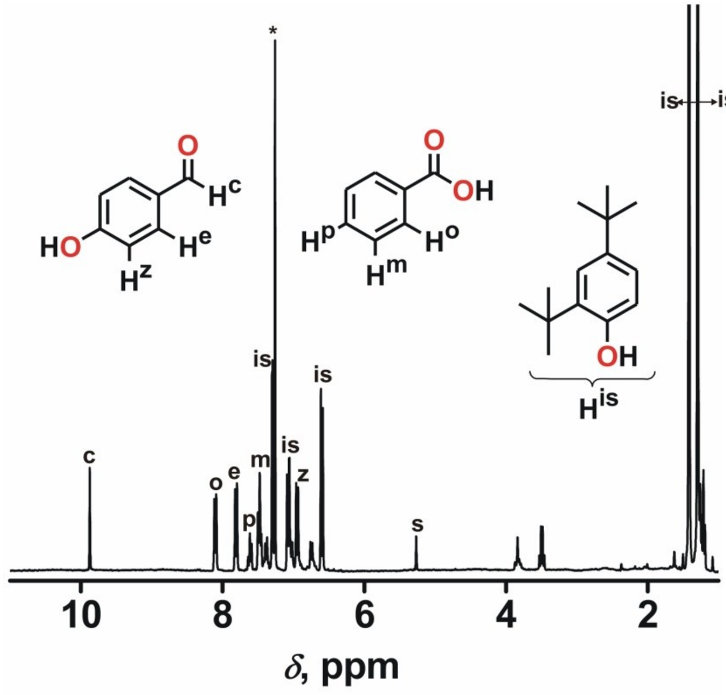

Fig. S27 ^{1}H NMR spectrum (500 MHz, $CDCl_3$, 295 K) of 4-hydroxybenzaldehyde formed in the reaction of **1** with O_2 in the presence of 4-hydroxybenzyl alcohol (10 equiv) in acetonitrile. Peaks marked with (is) are derived from 2,4-di-*tert*-butylphenol used as an internal standard.

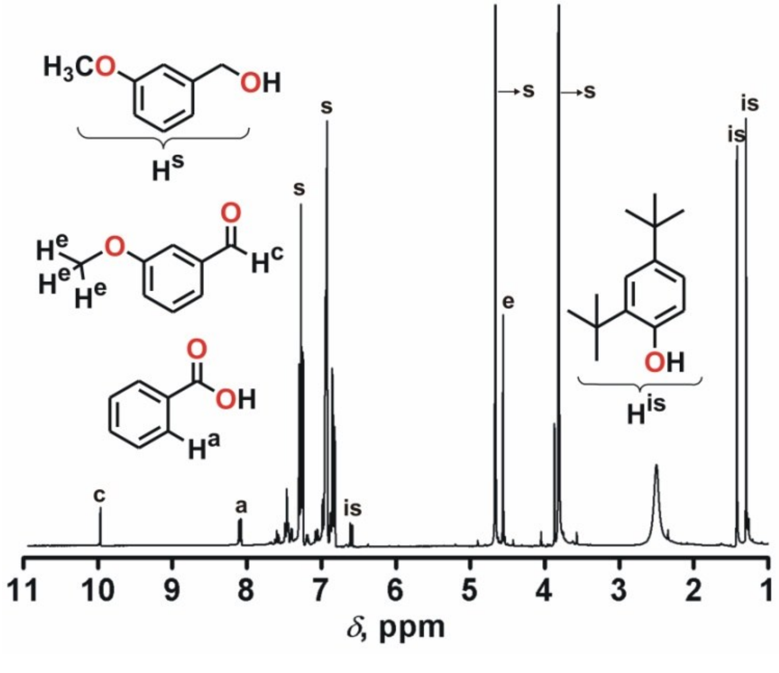

Fig. S28 ^{1}H NMR (500 MHz, $CDCl_3$, 295 K) spectrum of 3-methoxybenzaldehyde formed in the reaction of **1** with O_2 in the presence of 3-methoxybenzyl alcohol (10 equiv) in acetonitrile.

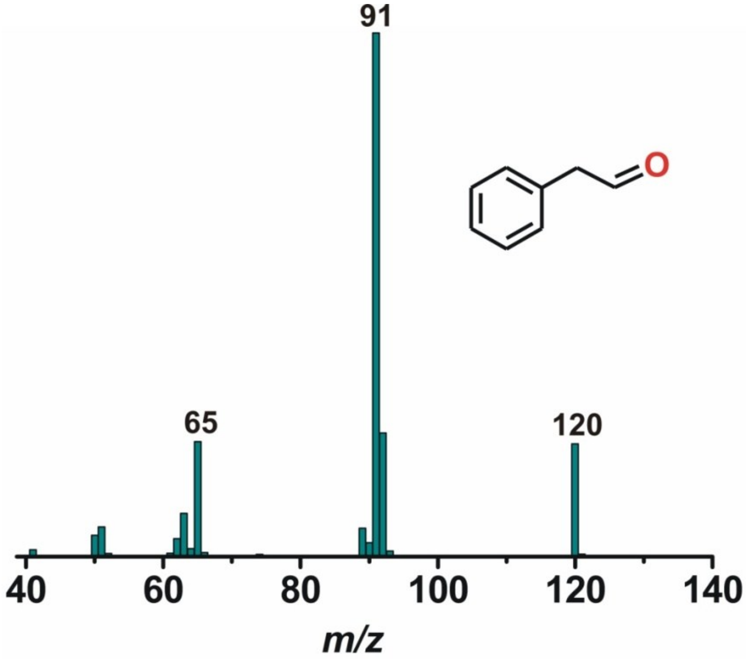

Fig. S29 GC-mass spectrum of phenylacetaldehyde formed in the reaction of **1** with phenethyl alcohol (20 equiv).

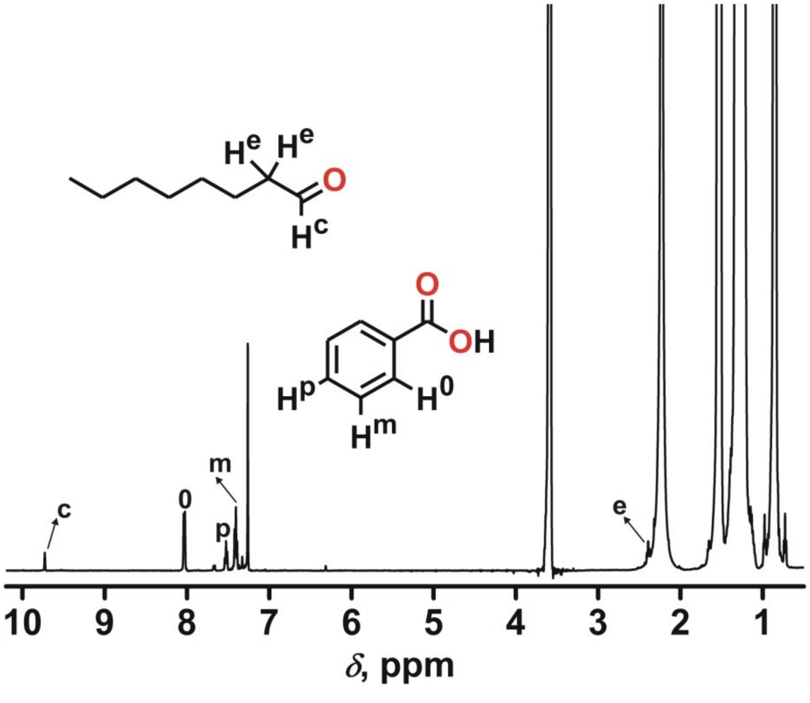

Fig. S30 ^{1}H NMR (500 MHz, $CDCl_3$, 295 K) spectrum of 1-octanal formed in the reaction of **1** with O_2 in the presence of n-octanol (10 equiv).

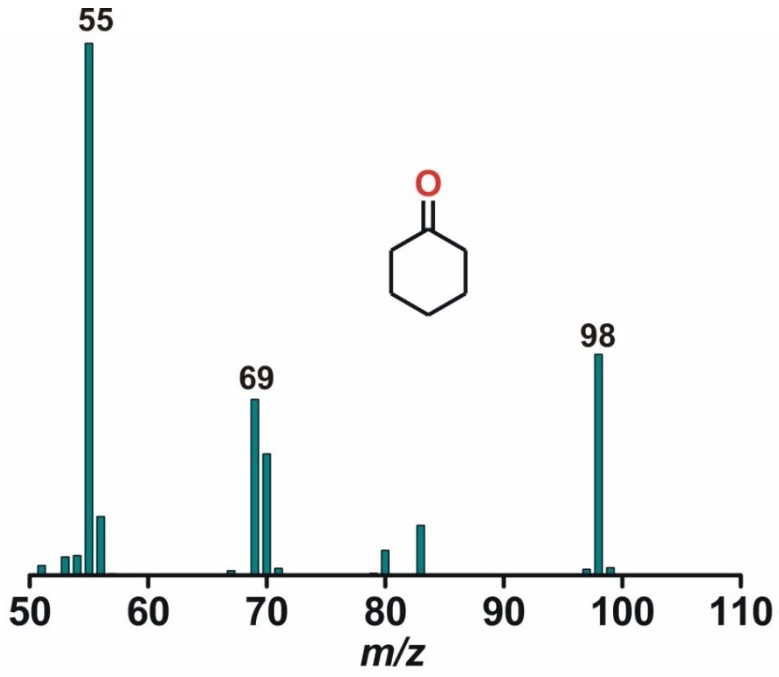

Fig. S31 GC-mass spectrum of cylohexanone formed in the reaction between **1** and O_2 in the presence of cyclohexanol (20 equiv).

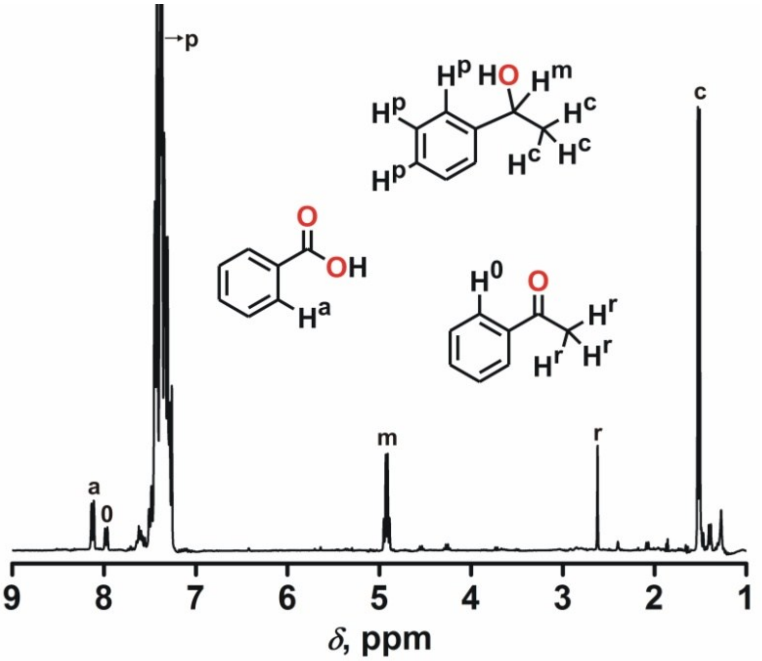

Fig. S32 ^{1}H NMR (500 MHz, $CDCl_3$, 295 K) spectrum of acetophenone formed in the reaction of **1** with 10 equiv of 1-phenyl ethanol in acetonitrile.

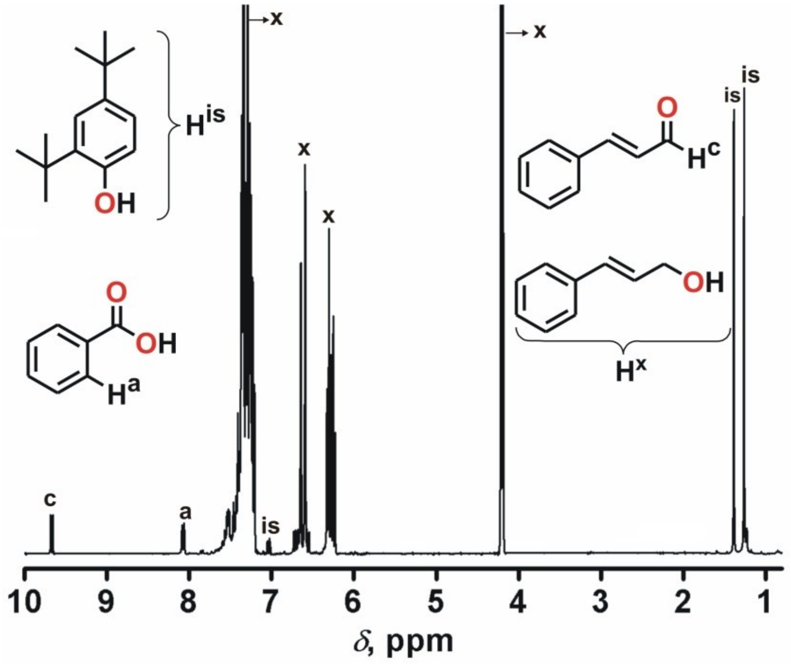

Fig. S33 ^{1}H NMR (500 MHz, $CDCl_3$, 295 K) spectrum of cinnamaldehyde formed in the reaction of **1** with O_2 in the presence of cinnamyl alcohol (10 equiv) in acetonitrile. Peaks marked with (is) originate from 2,4-di-*tert*-butylphenol used as an internal standard.

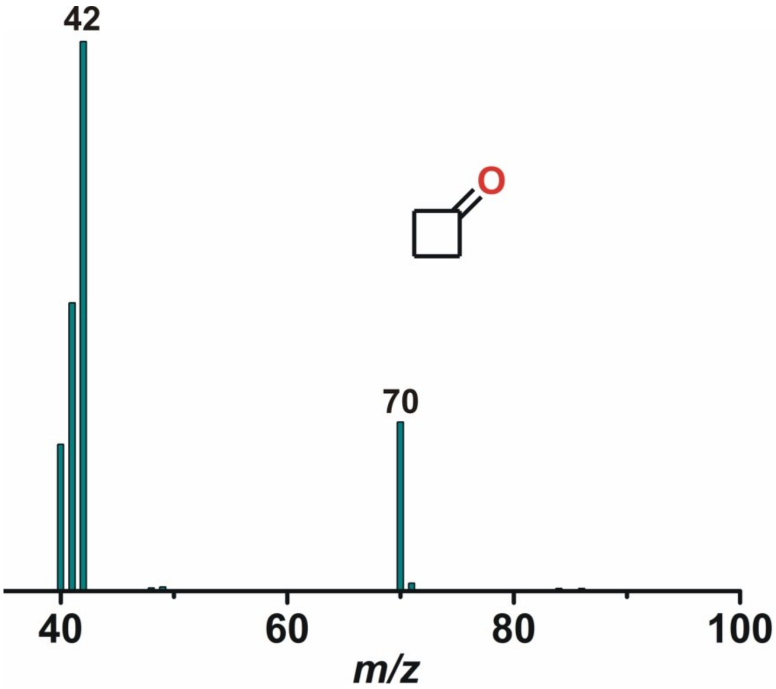

Fig. S34 GC-mass spectrum of cylobutanone formed in the reaction of **1** with cyclobutanol in acetonitrile.

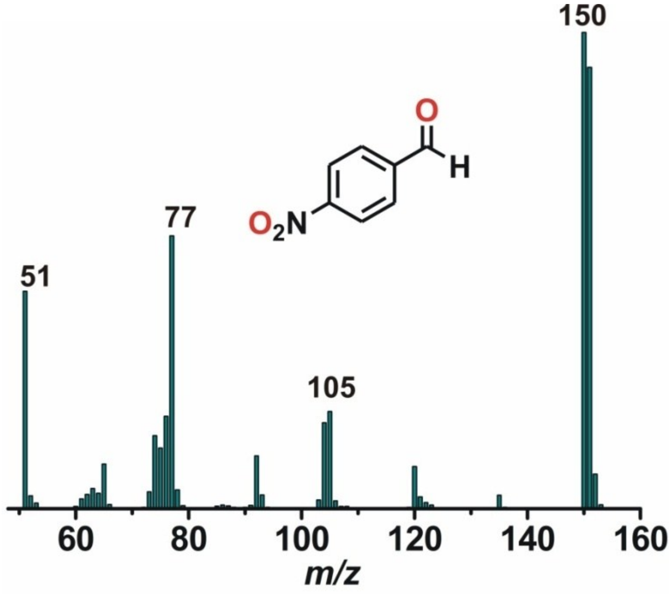

Fig. S35 GC-mass spectrum of 4-nitrobenzaldehyde formed in the reaction of **1** with $^{18}O_2$ in the presence of 4-nitrobenzyl alcohol.

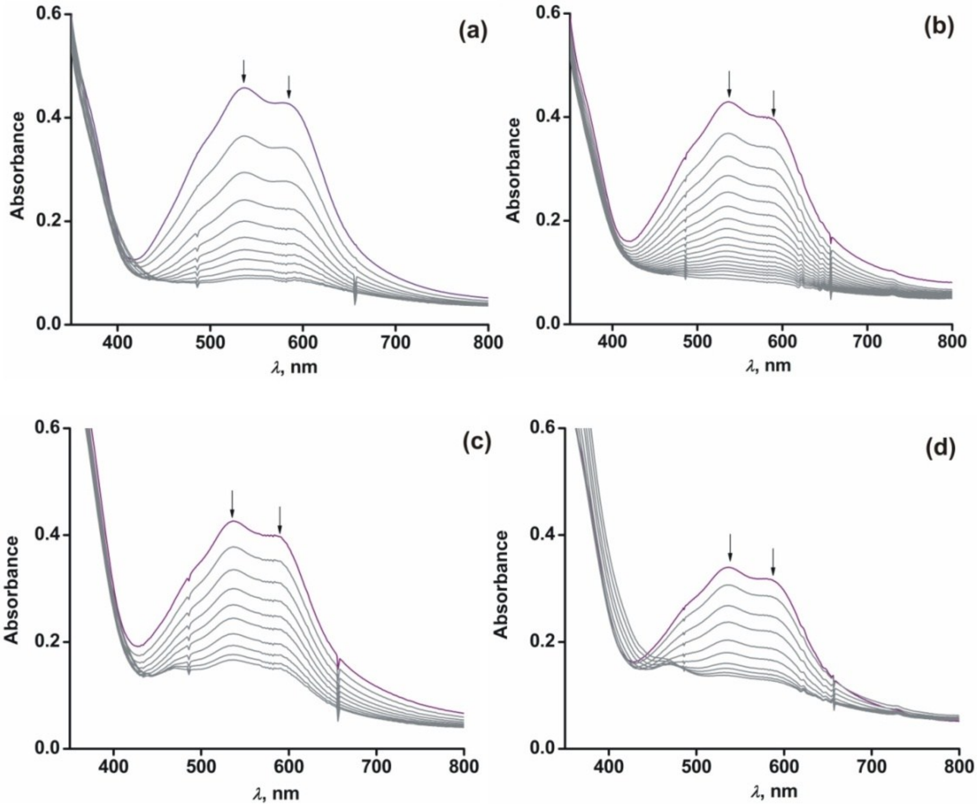

Fig. S36 Optical spectral changes during the reaction of **1** (1 mM) and benzyl alcohol (10 equiv) (a) with no added BF, (b) with 1 equiv of HBF+TEA (TEA = triethylamine), (c) with two equiv of HBF+TEA, and (d) with three equiv of HBF+TEA in acetonitrile.

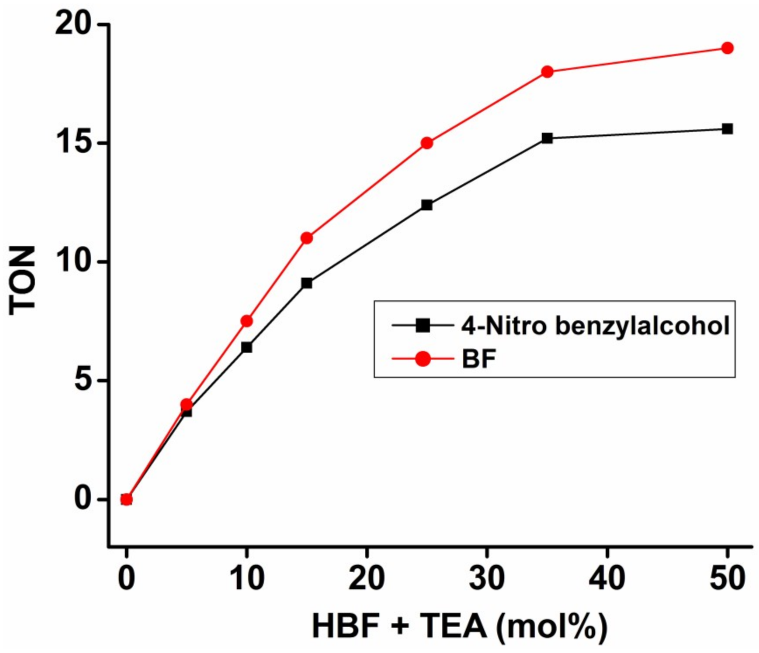

Fig. S37 Dependence of TON on the amount of HBF+TEA (1:1). Reaction conditions: 0.02 mmol catalyst (complex **1**) and 1 mmol (50 equiv) *para*-nitrobenzyl alcohol in acetonitrile for 8 h at 25 °C. TON for the formation of *p*-nitrobenzaldehyde from *p*-nitrobenzyl alcohol (black) and TON for the formation of benzoic acid from benzoylformate (red).

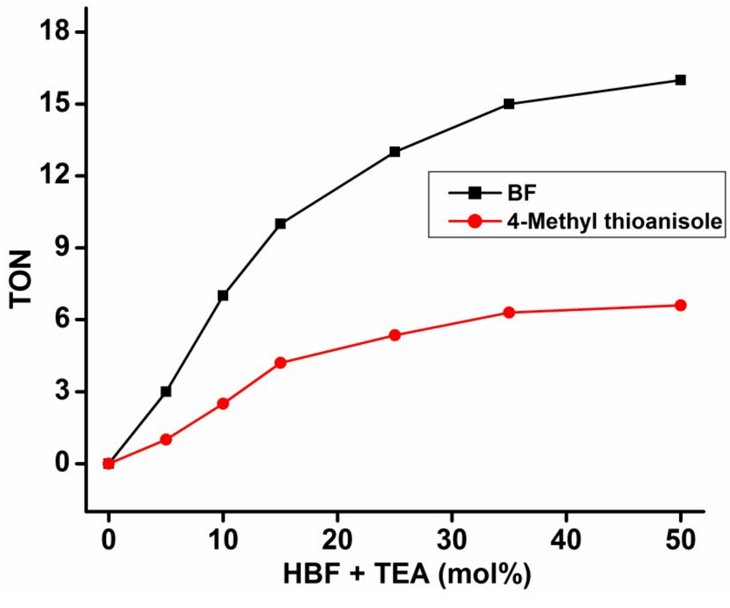

Fig. S38 Dependence of TON on the amount of HBF+TEA. Reaction conditions: 0.02 mmol catalyst (complex **1**) and 1 mmol (50 equiv) *para*-methyl thioanisole in acetonitrile for 8 h at 25 °C. TON for the formation of *p*-methyl thioanisole oxide from *p*-methyl thioanisole (red) and TON for the formation of benzoic acid from benzoylformate (black).

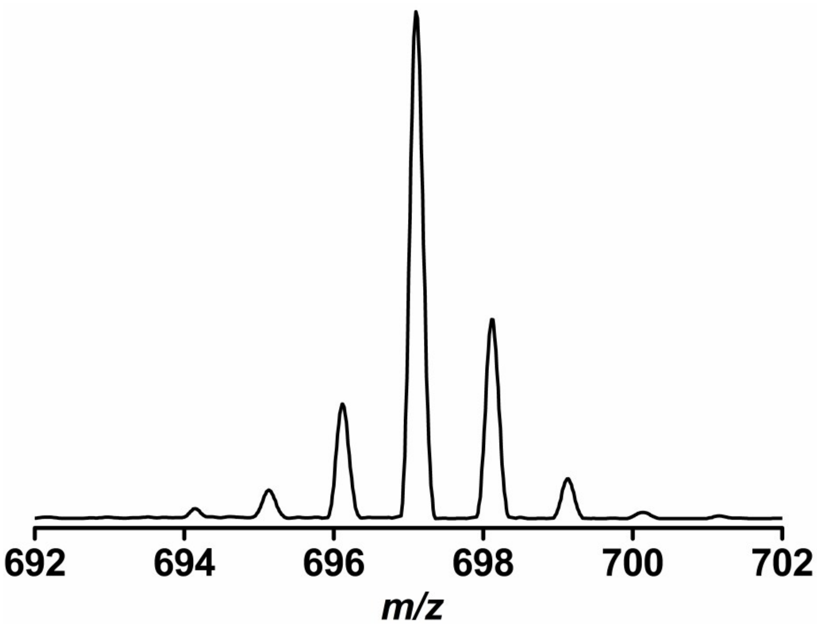

Fig. S39 ESI-mass spectrum (positive ion mode in CH_3CN-CH_2Cl_2) of the oxidized solution of **1** after catalytic reaction.

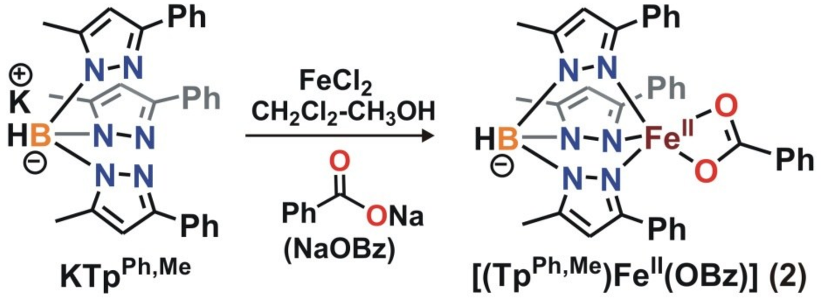

Scheme S1 Synthesis of iron(II)-benzoate complex **2**.

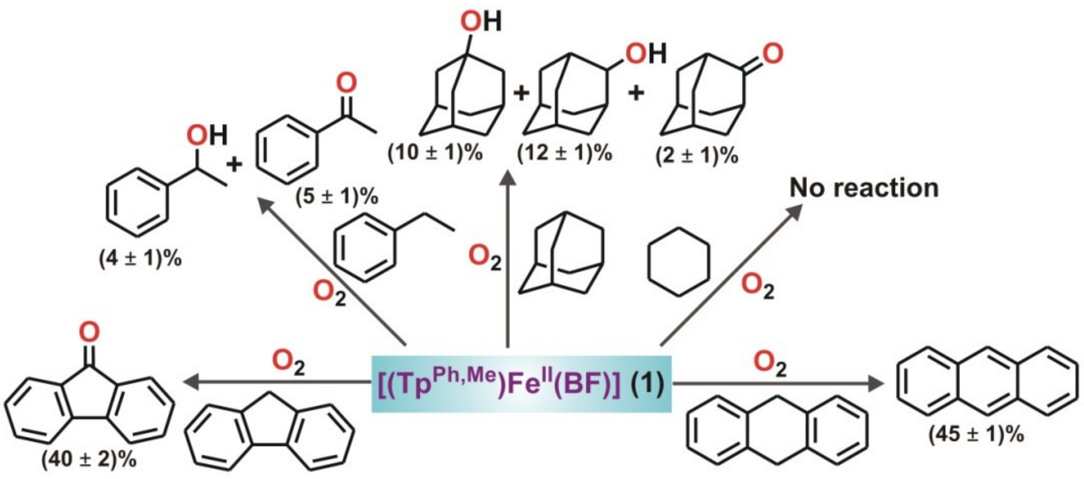

Scheme S2 Reaction of **1** with different hydrocarbons.

Table S1 Selected bond lengths (Å) and bond angles (°) for $[(Tp^{Ph,Me})Fe^{II}(BF)]$ (**1**) and $[(Tp^{Ph2})Fe^{II}(BF)]$.

	$[(Tp^{Ph,Me})Fe^{II}(BF)]$	$[(Tp^{Ph2})Fe^{II}(BF)]$
Fe1–N2 [N4]	2.083 (2)	2.068(5)
Fe1–N4 [N2]	2.209 (2)	2.188(5)
Fe1–N6 [N6]	2.065 (2)	2.086(5)
Fe1–O2 [O1]	1.957(2)	1.968(4)
Fe1–O3 [O2]	2.262(2)	2.206(5)
[N4] N2–Fe1–O2 [O1]	124.95(8)	132.6(2)
[N6] N6–Fe1–N2 [N4]	92.41(8)	91.1(2)
[N2] N4–Fe1–O3 [O2]	172.98(7)	171.7(2)
[N2] N4–Fe1–O2 [O1]	109.26(7)	110.1(2)
[N6] N6–Fe1–N4 [N2]	86.13(7)	89.0(2)
[N2] N4–Fe1–N2 [N4]	89.50(7)	86.4(2)
[N4] N2–Fe1–O3 [O2]	92.32(7)	85.7(2)
[O1] O2–Fe1–O3 [O2]	75.13(6)	77.3(2)
[N6] N6–Fe1–O2 [O1]	138.42(9)	131.5(2)
[N6] N6–Fe1–O3 [O2]	87.02(6)	88.5(2)

The atoms in the parentheses indicate the numbering of the atoms in $[(Tp^{Ph2})Fe^{II}(BF)]$ (M. P. Mehn, K. Fujisawa, E. L. Hegg and L. Que, Jr., J. Am. Chem. Soc., 2003, ***125****, 7828-7842) with the same geometry as* ***1****.*

Table S2 Selected bond lengths (Å) and bond angles (°) for [(Tp^{Ph,Me})Fe^{II}(OBz)] **(2^{PzH})**.

Fe1–N2	2.1412(13)	Fe1–O1	2.0047(12)
Fe1–N4	2.1179 (13)	Fe1–N8	2.1430(13)
Fe1–N6	2.1788 (13)	C1–O1	1.243(2)
C1–C2	1.522(2)	C1–O2	1.2554(19)
N4–Fe1–O1	109.31(5)	N8–Fe1–N6	168.97 (5)
N4–Fe1–N2	94.42(5)	N8–Fe1–O1	97.90(5)
N4–Fe1–N6	89.19(5)	N8–Fe1–N2	89.58(5)
N4–Fe1–N8	96.22(5)	N6–Fe1–O1	89.30(5)
N2–Fe1–O1	154.04(5)	N6–Fe1–N2	80.41(5)

…………….
